# Fungi-on-a-Chip: microfluidic platforms for single-cell studies on fungi

**DOI:** 10.1093/femsre/fuac039

**Published:** 2022-08-24

**Authors:** Felix Richter, Saskia Bindschedler, Maryline Calonne-Salmon, Stéphane Declerck, Pilar Junier, Claire E Stanley

**Affiliations:** Department of Bioengineering, Imperial College London, South Kensington Campus, Exhibition Road, London SW7 2AZ, United Kingdom; Laboratory of Microbiology, University of Neuchâtel, Rue Emile-Argand 11, CH-2000 Neuchâtel, Switzerland; Laboratory of Mycology, Université catholique de Louvain, Place Croix du Sud 2, B-1348 Louvain-la-Neuve, Belgium; Laboratory of Mycology, Université catholique de Louvain, Place Croix du Sud 2, B-1348 Louvain-la-Neuve, Belgium; Laboratory of Microbiology, University of Neuchâtel, Rue Emile-Argand 11, CH-2000 Neuchâtel, Switzerland; Department of Bioengineering, Imperial College London, South Kensington Campus, Exhibition Road, London SW7 2AZ, United Kingdom

**Keywords:** fungal biology, yeast, Fungi-on-a-Chip microfluidic technology, single-cell microscopy, arbuscular mycorrhizal fungi, fungal highways

## Abstract

This review highlights new advances in the emerging field of ‘Fungi-on-a-Chip’ microfluidics for single-cell studies on fungi and discusses several future frontiers, where we envisage microfluidic technology development to be instrumental in aiding our understanding of fungal biology. Fungi, with their enormous diversity, bear essential roles both in nature and our everyday lives. They inhabit a range of ecosystems, such as soil, where they are involved in organic matter degradation and bioremediation processes. More recently, fungi have been recognized as key components of the microbiome in other eukaryotes, such as humans, where they play a fundamental role not only in human pathogenesis, but also likely as commensals. In the food sector, fungi are used either directly or as fermenting agents and are often key players in the biotechnological industry, where they are responsible for the production of both bulk chemicals and antibiotics. Although the macroscopic fruiting bodies are immediately recognizable by most observers, the structure, function, and interactions of fungi with other microbes at the microscopic scale still remain largely hidden. Herein, we shed light on new advances in the emerging field of Fungi-on-a-Chip microfluidic technologies for single-cell studies on fungi. We discuss the development and application of microfluidic tools in the fields of medicine and biotechnology, as well as in-depth biological studies having significance for ecology and general natural processes. Finally, a future perspective is provided, highlighting new frontiers in which microfluidic technology can benefit this field.

## Introduction

The kingdom Fungi is a most intriguing one. For centuries, mycologists have been baffled by the vast diversity of fungi and struggled to assign them to Aristotle’s dual classification system of Plantae and Animalia. Only when Whittaker (Whittaker 1969) proposed the novel five-kingdom classification system in 1969, and advances in molecular genetics supported the classification (Kendrick 2017), did fungi obtain their status as an independent kingdom. Combining traits from each of the other kingdoms while having unique features, fungi share a heterotrophic lifestyle with animals, yet possess cells comprised of a cell wall, as well as vacuoles, and procreate via sexual and asexual reproduction just like plants. Moreover, the vast mycelium that many fungi form in the soil is reminiscent of the root system of plants, but with otherwise different dimensions. In fact, fungal mycelia have been found to span areas of up to 10 km^2^, such as mycelia of the basidiomycete *Armillaria ostoyae* (Schmitt and Tatum 2008), and can represent up to 50% of soil dry weight (Ingham et al. 1991). However, despite these somewhat macroscopic dimensions, fungi are considered as microorganisms, just like bacteria. Common features shared with bacteria include unique metabolic pathways, such as the very specific isomerization between homoaconitate and homoisocitrate (Fazius et al. 2012), as well as the microscopic dimensions of most fungi, especially yeasts, which are commonly unicellular. Filamentous moulds and mushrooms, however, are comprised of microscopic features, termed hyphae, which form extensive mycelia and prominent fruiting bodies on the macroscale (Webster and Weber 2007). Other species are dimorphic, meaning they can switch from one morphology to another (i.e. unicellular to filamentous). *Candida albicans* for instance, has several forms, including single-cell yeast as well as filamentous forms (Kadosh 2019). Along with this shape-shifting at the microscopic scale, some fungal species also express different morphologies whether they are in a vegetative, asexual, or sexual phase. Historically, this has led to the accidental naming of the same species more than once. This is particularly common in filamentous fungi. For some species, a dual nomenclature was kept, with different names given for the sexual and asexual stage or part of the fungus. Unprecedented in nature, this phenomenon led to the coining of the terms teleomorph (the sexual form), anamorph (the asexual form), and holomorph (fungi for which both a sexual and asexual lifestyle is known). However, due to much disagreement among mycologists and confusion regarding the dual nomenclature system, it was abandoned as of 1 January 2013 and replaced by the principle of one fungus having one name (‘one fungus, one name’; Wingfield et al. 2012). Of all the suspected 2.2–3.8 million fungal species in nature, it is estimated that only about 148 000 have been described so far (Hawksworth and Lücking 2017). Compared to the other fields of study in biology, mycology is a relatively young discipline and still considered a niche.

Until the last decade, mycology has regularly been considered as the study of mushrooms, i.e. the macroscopic structures that are found in forests or that we buy in a supermarket to eat. Other fungi, such as pathogens or symbionts (e.g. mycorrhizal fungi) have been handled in other fields, but not as integral parts of a larger and common area of research. As a result of this dispersion, their importance as a microbial clade has somewhat been underrepresented for many years. However, their significance is far reaching, and the relatively new fungal kingdom includes a vast number of species that are of the utmost importance for nature as a whole. Hence, not only are fungi fascinating and elusive, they also constitute an enormous significance for nature and humanity. Fungi act as the main decomposers in natural recycling processes and are responsible for degrading dead organic matter. Besides that, fungi are very potent biogeochemical engines, affecting the availability of numerous elements. This ability can be exploited artificially for bioremediation or in biofertilization (Carvajal-Muñoz and Carmona-Garcia 2012). In particular, the symbiotic association between a certain type of fungi and roots of plants—known as a mycorrhiza—is capable of significantly increasing yield in crops (Hamel 2007). Furthermore, fungal metabolic pathways can be harnessed for the biotechnological production of many important substances. Famous examples include the mass production of ethanol by the yeast *Saccharomyces cerevisiae*, with almost 110 billion litres produced annually, as estimated by the U.S. Department of Energy (2020), or the production of citric acid by the mould *Aspergillus niger* (Show et al. 2015). Medically relevant examples include the antibiotic penicillin (Barrios-González et al. 1988) and the immunosuppressant cyclosporin A (Ramana Murthy et al. 1999). On the other hand, however, some fungi are responsible for causing severe diseases in humans (e.g. *Cryptococcus neoformans* or *Aspergillus fumigatus*) and other animals, but also in plants. The pathogen *Fusarium graminearum*, for example, is responsible for annual losses in wheat and barley production ranging between 6% and 19% in the USA, which translates to a loss on the order of billions of dollars (McMullen et al. 2012). Due to a lack of effective antifungal agents (Almeida et al. 2019), promising alternatives to the use of chemical fungicides are being developed, including biocontrol agents such as the mycoparasitic fungi *Clonostachys rosea and Trichoderma* spp. However, more basic research into their infection mechanisms at the cellular and intracellular levels is needed to eventually harness their antifungal potential in a widespread, commercial way.

So-called microfluidic—or ‘Fungi-on-a-Chip’—technologies are emerging as a new tool to aid such studies in mycology, as well as high-throughput applications in fungal biotechnology. The field of microfluidics involves the manipulation of small amounts of liquids in a controlled manner within artificial micron-sized fluidic networks of channels. It was established as a useful tool for different kinds of studies, including nanoparticle synthesis (Karnik et al. 2008), general chemical synthesis (Liu and Jiang 2017), and chromatography (Xie et al. 2005). The original concept stemmed from a need to implement different automated operations (e.g. sorting capabilities) into chemical processes, in an analogous manner to that of electrical networks, whilst increasing experimental throughput concomitantly. Due to the significantly smaller dimensions of micron-sized fluidic networks, other effects dominate when compared to the macroworld. Hence, a transition to miniaturized systems yields new experimental opportunities. Above all, the flow behaviour within microchannels is mostly laminar, having a parabolic streaming profile described by the law of Hagen and Poiseuille (Sutera and Skalak 1993). While gravity is widely negligible, capillary forces and surface tension dominate, allowing new possibilities to be exploited such as droplet trapping or inertial microfluidics. Generally, miniaturization enables the generation of high-throughput experimental systems that possess enhanced analytical accuracy and sensitivity, which is closely interlinked with the ability to control processes in an outstandingly precise manner, especially in terms of temperature, illumination, or flow dynamics. Moreover, this entails a safer handling, as well as intrinsic sterility, because of the enclosed nature of the microsystem. While the requirements for chemical or biological material, both in terms of quantity and cost, are usually significantly lower than those associated with conventional methods (e.g. batch processes), it is still possible to achieve high output of both results or products by following a modular approach and parallelization, also called numbering-up (Kriel et al. 2016).

In recent years, microfluidic technologies have been adopted for applications in the biological sciences and in particular for studies on whole (living) organisms. This trend is evidenced by the many advantages that microfluidic platforms offer, such as the optical transparency of most devices for brightfield and fluorescence-based imaging as well as the ability to mimic microenvironments both structurally and with well-defined chemical gradients. Material choice is key for the desired application, with the elastomeric polymer, poly(dimethylsiloxane) (PDMS) featuring as one of the most prominent materials used in microfluidic technology development (Box 1). In combination with high-throughput and automated approaches, microfluidic technologies are becoming increasingly recognized by biologists as experimental platforms that can afford precise analyses on a cellular, subcellular, and even molecular level, in genetics, proteomics, and cellular assays (Tian and Finehout 2009). The latter is especially well-represented in the literature, with numerous examples of studies with bacteria (Cao et al. 2013) and human cells (mainly blood, sperm, or stem cells; Zhang et al.2018a). In particular, microfluidic technology has been extended to the study of small multicellular organisms, e.g. nematodes (Lockery et al. 2008, Tayyrov et al. 2019), as well as mammalian tissue (Zheng and Jiang 2018), plants (Stanley et al. 2018), and animals (Funfak et al. 2007). Microfluidic studies involving fungi, however, are rare. Indeed, the application of microfluidics to mycology is a very recent development with the first studies published only in the last 15 years. Indicative of this, Tian and Finehout’s book ‘Microfluidics for Biological Applications’ published in 2009 (Tian and Finehout 2009) gives a very detailed overview about biological investigations using microfluidic methods, without, however, a single mention of fungi.

Box 1. **Materials used in microfluidic technologies**

As the field of microfluidics originated from microelectronics, silicon was one of the first materials to be used in microfluidic device development. Although having well-defined material characteristics and well-established manufacturing processes, silicon was soon replaced by glass. Glass possesses important properties, such as chemical inertness, mechanical resilience, and optical transparency, as well as the opportunity to etch micron-sized channel architectures. This, coupled with the overall good biocompatibility of glass, makes it a suitable material for the use in microscopy investigations of microorganisms and biological processes. With similar properties, but generally much easier, safer, and quicker to fabricate using lithography methods, polymers have mostly succeeded glass in microfluidic technology development. Especially the thermoplastics such as polycarbonate (PC; Ogończyk et al. 2010), poly(methyl methacrylate) (PMMA; Hong et al. 2010), cyclic olefin copolymer (COC; Ochs et al. 2014), as well as polystyrene (PS; Young et al. 2011) are widely used. The microchannel layout is commonly engraved via hot embossing, precision milling, or laser ablation. Even though all of these materials have their benefits and applications, the elastomeric polymer poly(dimethylsiloxane) (PDMS) has emerged as one of the main materials of choice in microfluidic device fabrication. The majority of the studies discussed herein feature PDMS as the basis for their microfluidic devices. PDMS can be cast easily from master moulds using soft lithography, with little apparative requirements. Besides the uncomplicated device manufacturing, PDMS offers optical transparency, low autofluorescence, gas permeability, adjustable tensile strength, and exceptional biocompatibility (Raj and Chakraborty 2020). The elasticity of PDMS allows to form valve and pump structures (Nielsen et al. 2020) or to measure forces exerted by a microorganism, however, this also renders PDMS devices unsuitable for high-pressure tasks.

Besides these established methods, other materials have emerged for specialist applications. This includes smart materials, such as pressure-controlled adhesives (Nielsen et al. 2020) and certain hydrogels (Kang et al. 2013), the latter of which combines structural with functional properties. Similarly, ceramics are being used for microfluidic devices, contributing excellent thermostability and thermal insulation as well as chemical stability to the system (Vasudev et al. 2012).

Herein, we review the major studies conducted within the emerging field of Fungi-on-a-Chip microfluidic technology development for single-cell studies on fungal biology. First, we highlight the application of microfluidics for investigations on yeast cells, the first type of fungi to be featured in microfluidic systems. The collected publications are mainly dedicated to investigations on cellular life cycle and ageing, as well as the design of trapping facilities for single-cell capture. Through these single-cell studies, it was found that cellular responses and expression levels vary immensely from cell-to-cell, far more than previously expected and independent from the cell cycle stage. These findings will be important for improving the effectiveness of biotechnological processes and yield new hypotheses related to cell ageing studies, moving away from investigations at the population level to individuals. In the second major section, we discuss a range of medical applications, mainly blood cleansing devices for fungal pathogens, as well as Lung-on-a-Chip models for fungal disease studies. Highlights of this section include a microfluidic device that revealed an altered immune response in bronchiole models when coinfected with fungi and bacteria, compared to infection with both monocultures alone, an insight key to understanding dangerous diseases like cystic fibrosis. The third section is dedicated to investigations concerned with filamentous fungi and their interactions with other organisms within artificial microfluidic devices at the cellular level, which has formed a significant trend of late, especially in the emerging field of Soil-on-a-Chip microfluidics (Stanley et al. 2016). Using a microfluidics-based approach, microenvironments can be designed to allow environmental cues of a chemical, physical, and biotic nature to be studied in depth, while retaining transparency for detailed analyses, e.g. using high resolution microscopy techniques. For instance, the pioneering work by Held et al. (2009a, 2011a) unravelled the space searching strategies of the filamentous fungus *Neurospora crassa*, which is directed by the Spitzenkörper, an intracellular body that acts as a gyroscope for directional memory and manoeuvring coordination. Lastly, we provide a future perspective detailing a number of new frontiers in which microfluidic technology is expected to aid future research in this field of study, with a focus on arbuscular mycorrhizal fungi (AMF), nuclei behaviour in filamentous fungi, multispecies interactions, and electrical measurements for cell-to-cell communication.

## Single-cell studies on yeast

The first type of fungi to be featured in a microfluidics-assisted investigation were yeasts. Because of their single-cell character, the transition from microfluidic studies on bacteria and mammalian cells was a smooth one, often starting with simply extending the application of established devices to the yeasts. Wu et al. (2004) designed the first microelectromechanical systems (MEMS)-based device involving yeast cells, which allowed single cells to be aligned in a row and subsequent fluorescence-based imaging and counting of individual cells. Due to a channel width of 7 µm and sheath flow-assisted focussing, only one cell at a time was able to pass through the channel network and be admitted into the image identification unit. Their simple glass-based microdevice comprised of an inlet for the cells, specifically *S. cerevisiae* and prostate cancer cells of the PC3 line, two channels to generate the sheath flow and three outlets with vacuum suction. In the study, the general functionality of the device was proven and further applications such as cell separation were proposed, marking the starting point of fungal microfluidics. Moreover, the authors proclaimed that the set up and entailing shear stress, as well as pressure, did not pose a negative impact on the cells, an issue debated in later studies.

### Imaging microcolonies

**Figure 1.**
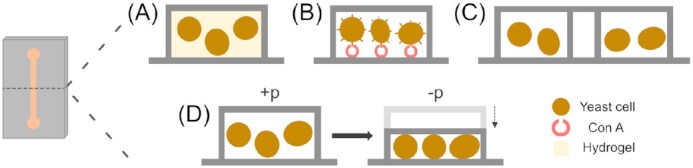
Schematic illustrating yeast cell immobilization strategies. The illustration shows cross-sections through a microchannel to describe different methods of immobilizing yeast cells in a microfluidic device. (A) Cells fixed by *in situ* polymerization of a hydrogel. (B) Yeast cells fixed by affinity binding using Concanavalin A. (C) Spatial segmentation of cells into microcolonies via passive trapping. (D) Pressure-based trapping of cells between the channel ceiling and bottom. When flow pressure is high (+p), the channel widens and cells enter into the microchamber. Upon release of pressure (-p), the channel shrinks to a size comparable to that of the cell diameter, thus immobilizing the cells.

Yeasts, especially the model organisms *S. cerevisiae* and *Schizosaccharomyces pombe*, are of the utmost importance to researchers. These unicellular fungi are eukaryotic entities with close resemblance to mammalian cells, making them perfect subjects for detailed studies concerning unravelling the function of every gene in the genome, as well as investigations on gene expression, intra- and intercellular signalling and replicative cell ageing (Dolinski and Botstein 2005, Pardo et al. 2016, Piper 2006). These processes occur across various microscopic scales, ranging from the microcolony level, to the level of single cells, and down to the subcellular level, for which microfluidic technologies are ideally suited. It is unsurprising, therefore, that numerous groups have integrated microfluidic techniques into their studies with yeast. Microfluidic devices allow cells to be aligned within one focal plane for precise imaging, as well as providing a means to separate them into different chambers (i.e. to conduct experiments in parallel) and afford highly controlled perfusion with effector solutions. The exact device designs can be then tailored to the task at hand. Several methods for the trapping of yeast cells within microfluidic devices have been employed to date, since yeasts—unlike many mammalian cells—generally do not adhere tightly to surfaces and, therefore, need to be fixed. Figure 1 highlights the major methods employed to immobilize yeast cells, which includes *in situ* polymerization, affinity binding, passive trapping in microchambers, and pressure-based active trapping. Some of these methods/techniques used for cell ageing-related studies have been reviewed by Chen et al. (2017) and Jo and Qin (2016).

#### In situ polymerization

A suitable means to accommodate yeast cells within a microfluidic channel is to immobilize them within a hydrogel, e.g. alginate (Vo et al. 2020) or agarose, which is a nontrivial task. The most used method, termed *in situ* polymerization (Falconnet et al. 2011), utilizes a low melting temperature liquid agarose gel, i.e. prepared and premixed with the cell suspension at 30°C. Once pumped into the designated chambers, the gel–cell mix is polymerized at 4°C for 3 min and the cells are robustly fixed. The sophisticated channel design and computer-assisted chemical control allows 128 parallel experiments to be conducted in a single run, whereby eight different *S. cerevisiae* genotypes and 16 different conditions can be probed simultaneously to investigate lineage-dependent pheromone signalling responses. While the results obtained reveal response dynamics on the single-cell level, the data is still statistically relevant. To demonstrate the potential of their device, the group conducted experiments of the mitogen-activated protein kinase (MAPK) involving mating responses towards different concentrations of the pheromone α-factor in different strains. As a read-out, they tagged the membrane receptor Ste2 with green fluorescent protein (GFP). Ste2 is up-regulated in the sexual tip extension of a yeast cell, called shmoo, and thus correlates with an enhanced mating activity, triggered by the pheromone gradient. The pheromone response found was highly variable from cell to cell. As an explanation, they suggested nongenetic inheritance of the behaviour since the yeast colonies were genetically identical.

**Figure 2.**
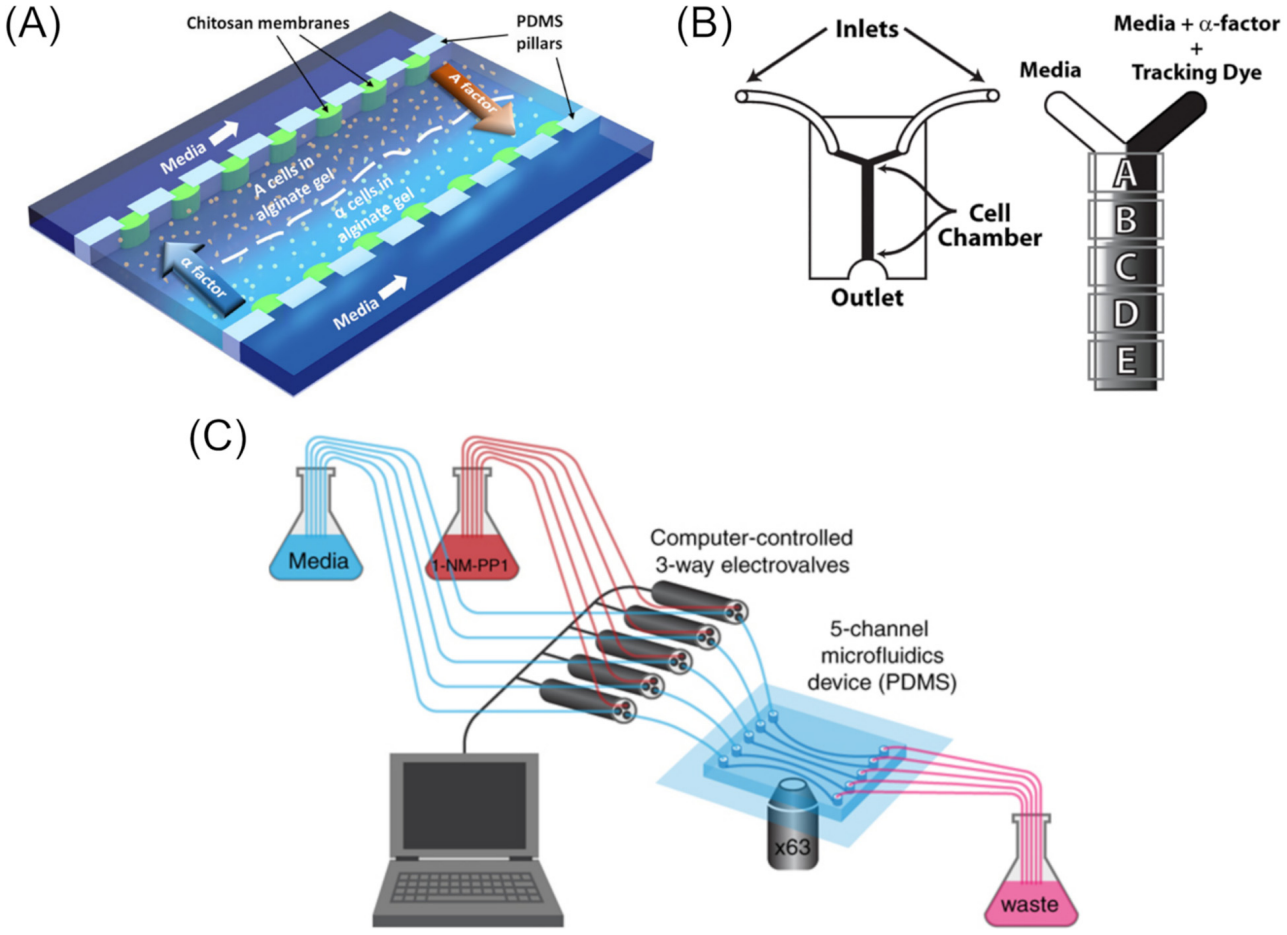
Examples of applications of microfluidic immobilization of yeast cells by *in situ* immobilization and affinity binding. (A) Device for studying pheromone chemotropism in α- and A-yeast cells. Cells are trapped in alginate gel and subjected to asymmetric pheromone conditions. Image reproduced with modifications from Vo et al. (2020) with permission from AIP Publishing (Licence Number: 5271490260805). (B) Y-device, coated with concanavalin A (Con A) to help α-cells adhere to the channel. A gradient of α-factor was created, followed with the aid of Dextran-3000-TRITC as a tracking dye. Cells in five different areas, A–E, were imaged. Image reproduced with modifications from Moore et al. (2008) with permission from the Creative Commons Attribution license (www.creativecommons.org/licenses/by/4.0/). (C) Example of five-channel device with computer-controlled 3-way valves to subject Con A-immobilized yeast cells to pulsed treatments with the protein kinase A (PKA) inhibitor 1-NM-PP1. A 63X microscope objective was used to monitor Msn2-mCherry translocation dynamics and gene expression in single cells. Image reproduced with modifications from Hansen and O’Shea (2013) with permission from John Wiley and Sons (Licence Number: 5271490732299).

The same experimental set up (with slight modifications) was used in further investigations probing the nongenetic heritability aspect of these pheromone response phenomena. The microfluidic design allowed to precisely track budding and thus create lineage maps within the microcolonies; the mother–daughter correlations in response to the α-factor pheromone differing from strain to strain could then be measured. Using a sophisticated tripartite fluorescent tagging strategy and targeted cell-cycle synchronization with hydroxyurea, the authors were able to reveal cell-to-cell variations in the cellular response, which would otherwise have been concealed within the colony. Moreover, it was found that the kinase Fus3 not only supresses the transition from the G1 to the S phase upon pheromone stimulation, but also modulates the cell cycle into the other direction, resulting in growth arrest preferably at the S phase and generally longer cell cycle phase duration in the Fus3 knockout mutant (*fus3Δ*), even without the pheromone stimulus. These findings suggest a stronger influence of cell cycle and the interplay between signalling pathways onto colony heterogeneities than formerly expected (Ricicova et al. 2013). Figure 2(A) shows a schematic experimental design utilizing hydrogel cell immobilization.

#### Affinity binding

Another method employed to immobilize yeast cells within the vicinity of the microchannel surface involves coating the channel with biomolecules, such as concanavalin A (Con A) or avidin (Xie et al. 2012). Con A, a lectin well-known for its application in affinity chromatography (Hage 1999), can bind specifically to several carbohydrate structures. In an analogous manner, Con A can bind to certain sugars presented on the surface of yeast cells, thus acting as an immobilizing agent. In comparison to agar, agarose and other hydrogel immobilization techniques, the use of Con A is claimed to cause minimal (or no) background fluorescence in microscopy (Pemberton 2014). Moore et al. (2008) demonstrated that a very simple chamber design could be employed, when Con A was used to prevent yeast cells from being flushed out. With their Y-shaped device design and using a relatively low volumetric flow rate, they generated a concentration gradient comprised of α-factor mating pheromone, which, under a laminar flow regime, relies on diffusion only. As a result, the gradient was the least steep closest to the outlet (Fig. 2B). The yeast cells then responded to the different concentrations of α-factor within the gradient, exhibiting different development of their mating projections (shmoo; i.e. involving directional growth strategies and error correction). Owing to the successful immobilization of single yeast cells, it was also possible to reveal the subcellular mechanisms underlying these behaviours.

A similar channel design was employed by Hersen et al. (2008), equipped with an electrovalve to create effector gradients more precisely and allow for oscillations of up to a few hertz (Hz). With this device, the cellular response of *S. cerevisiae* to environmental stresses was studied. To investigate the hyperosmolar glycerol (HOG) pathway, which involves MAPKs, a sorbitol solution was introduced to create osmotic stress. An increase in the osmotic pressure was followed by translocation of the MAP-Kinase Hog1 to the nucleus preceded by phosphorylation. If the stimulus frequency applied was lower than 4.6 × 10^–3^ Hz, the cellular response was found to follow the input oscillation instantaneously. Their findings suggested that the factor limiting the processing speed was the time required for the biochemical cascades to occur (e.g. phosphorylation) rather than physico-chemical mechanisms, which occur on much faster time scales (Hersen et al. 2008). The same device was later used by Hao and O’Shea (2011). In this study that used various stressors, such as potassium chloride, hydrogen peroxide, and high glucose concentrations, a deeper insight into the different kinetics of transcription factor-dependent cell responses was gained. It was shown that the transcription factors Msn2 (a general stress-responsive transcription factor in *S. cerevisiae*) and Crz1 (a zinc finger protein) are activated differently by these stimulants and that these patterns again trigger a specific promoter, thus encoding the input signal and translating it into an adequate stress response of the cell. More specifically, it was found that with slow promotors and high frequency input signals a temporal summation or ‘head-start’ effect can be caused, leading to a nonlinear relationship, while genes with rapid promotor kinetics are able to follow the input proportionally. At the molecular level, this can mainly be attributed to promotor structure, number, and position of binding sites and, thus the binding affinity. The idea of encoding or integration of environmental stresses into single molecule actuators (i.e. transcription factors) was further elaborated in a subsequent study conducted by the group (Hao et al. 2013). To elucidate the mechanism of the promotor activation and intrinsic amplitude thresholds, the group upgraded their microfluidic device from a single-channel to a five-channel architecture (Fig. 2C). With the additional throughput, they found trade-offs between the level of control regarding gene expression and expression noise. Thus, this work reiterates how different promotors can be seen as modules of signal-processing, with simple integrators, high-pass filters, and duration filters (Hansen and O’Shea 2013, Hansen et al. 2015). These collective studies suggest that the response can be specifically tuned to environmental factors through evolution of the promoters, for instance, a strategy that could be similarly applied to biotechnology.

#### Passive trapping in microchambers

**Figure 3.**
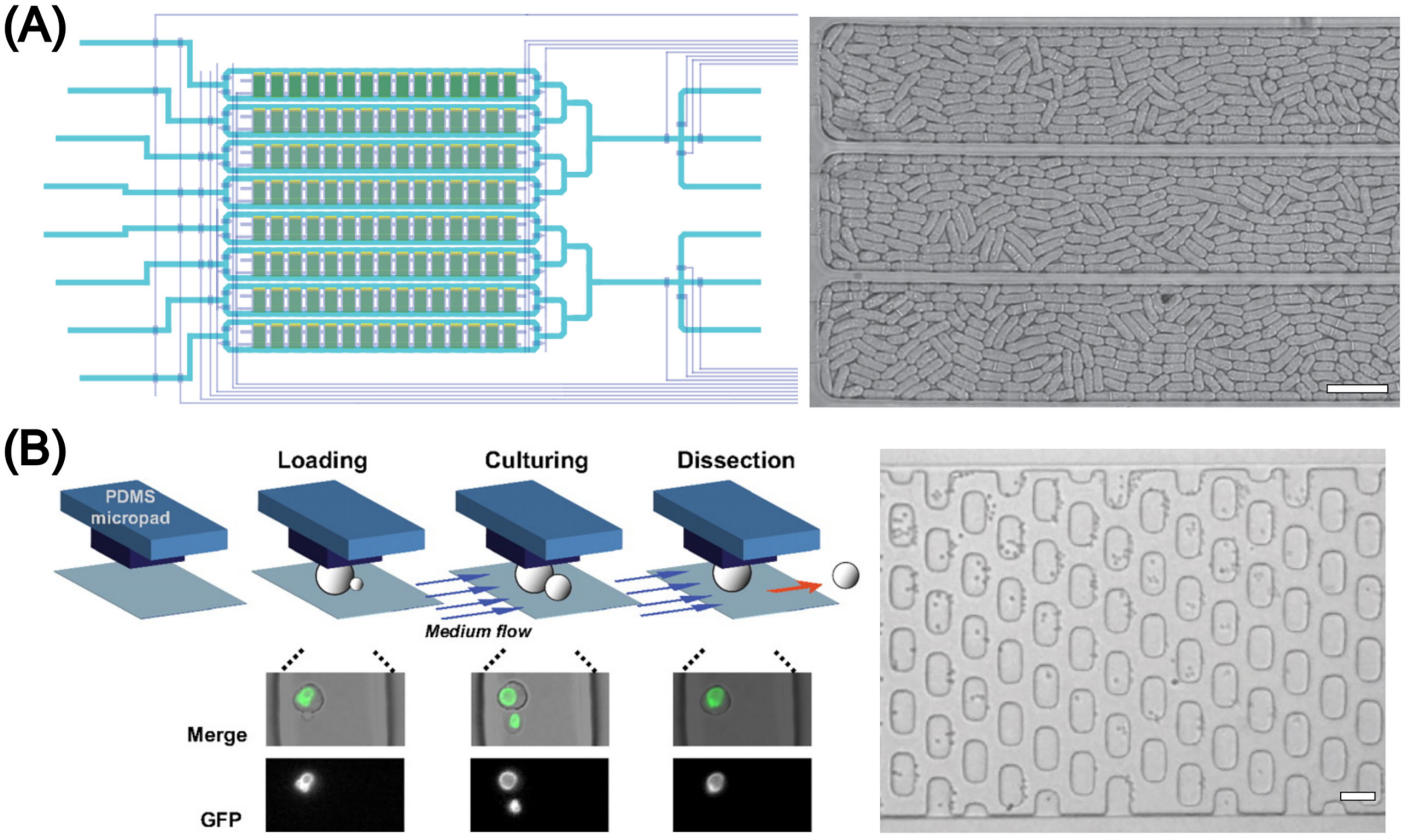
Exemplar applications of microfluidic immobilization of yeast cells by compartmentalization into microcolonies and active, pressure-based trapping. (A) Schematic illustration of a device having eight independently addressable rows each containing 15 microchemostats, highlighting the high degree of parallelization. Yeast microcolonies (here *S. pombe*) can be imaged and studied in parallel as well as subjected to different media conditions. The bright-field image shows three microchambers filled to confluence with yeast cells. Image reproduced with modifications from Nobs and Maerkl (2014) with permission from the Creative Commons Attribution license (www.creativecommons.org/licenses/by/4.0/). (B) Microfluidic platform for trapping single yeast cells (here *S. cerevisiae*) under pressure-based expandable micropads (30 × 15 µm), used to study cell ageing. During cell loading, the micropads are slightly lifted due to the hydrodynamic pressure created by the flow. Upon release of pressure, the micropads resume their original height, thus trapping cells. Smaller daughter cells budding off from the mother cells are automatically washed away during dynamic cultivation with a slow constant flow. Microscope image showing some yeast cells (mother cells) trapped underneath the micropads and some smaller cells (daughter cells) being flushed out. Image reproduced with modifications from Lee et al. (2012) with permission from National Academy of Sciences. Scale bars represent 20 µm.

The aforementioned techniques involved fixing yeast cells in gels (e.g. agarose) or with attachment agents (e.g. Con A) to provide immobilization. However, these approaches are claimed to affect the imaging quality of the yeast cells in a negative manner, e.g. by blurring the image or by introduction of crystals in case of incomplete polymerization, cells lose attachment after only a few hours (Ryley and Pereira-Smith 2006, Crane et al. 2014) and the cells themselves may also potentially be influenced with regard to their growth and development. Therefore, many groups set out to develop microfluidic devices to ‘cage’ cells, with microchambers able to hold the cells due to a specific channel design only, deciding to accept the trade-off between a less robust fixing of the cells with the added benefit of unaffected imaging and cell development. An example of a microfluidic device featuring an array of microchambers can be found in Fig. 3(A). Cookson et al. (2005) designed a simple, accessible microfluidic device loosely inspired by Nikola Tesla’s diode loop (Tesla 1920) to trap microcolonies of budding yeast. Using their channel design, they were able to assemble the cells in a single focal plane to produce high-resolution images. Time-lapse fluorescence microscopy was employed to track single cell cycles as far as 10 divisions in a dynamic manner, gaining statistical data for predictive modelling (Cookson et al. 2005).

Paliwal et al. (2007) devised a more complex device design, which utilized small chambers for perfusing microcolonies of *S. cerevisiae* cells with effector-media solutions on two sides. After loading the cells into the device there was no active flow inside of the cell chambers and the effector gradient reached the cells by diffusion only, preventing the cells from being flushed out. Here, and continued by Hao et al. (2008), morphological changes in yeast cells as a mating response to the pheromone gradients were investigated, similar to Moore et al. (2008), but more emphasis was put on the regulatory pathways involved, including the signalling components Fus3, Ste5, and Kss1. The ability to subject cells to different concentration gradients of pheromones more closely simulates reality, as compared to uniform concentrations commonly applied in standard, plate-based assays. Thus, the authors revealed the function of Ste5 in the stimulation and control of Fus3 as the main mechanism for gradient sensing and directional growth of the mating projection, as part of a complex interplay of all the signalling components. Interestingly, as a result of imaging with single-cell resolution, it was further discovered that a differentiated response occurs upon exposure of the microcolony to low pheromone levels, where some cells induce shmoo formation whereas others actively suppress this expression programme. Thus, they are effectively saving energy while waiting for the signal to become either stronger or subside (Hao et al. 2008, Paliwal et al. 2007). In an analogous manner, Taylor et al. (2009) studied pheromone mating responses in *S. cerevisiae*. Using their microfluidic device they achieved an immensely high analytical throughput, allowing them to take over 49 000 images of parallel experiments in just one run (∼12 h). Besides Fus3 and Kss1, they further extended pheromone response studies onto the signalling proteins Ptp2 and Msg5.between the different signalling

Denervaud et al. (2013) built a microfluidic device able to accommodate more than 10 000 separate experiments, observing 1152 GFP-expressing yeast strains in one run. This set up allowed dynamic investigations on changing yeast proteomes to be conducted in response to stressors such as ultraviolet (UV) radiation, methyl methanosulfonate, hydroxyurea, and osmotic stress, in a pulsed and continuous manner, helping to unravel the full dynamics of changes, which are often transient. It was elucidated that the P-body components Scd6 and Pat1 are predominantly involved in the formation of the mRNA processing body (P-body). Moreover, it was found that four main proteins (Tsa1, Rtt101, Rai1, and Nam7) are involved in nuclear localization of the ribonucleotide-diphosphate reductase Rnr4. The advantage of the presented method is that it facilitates the analysis of cellular signalling and expression mechanisms in combination with real-time phenotypical changes, a read-out that conventional methods such as spot assays are lacking. A slightly modified microfluidic device was later used by Nobs and Maerkl (2014) to study *S. pombe*. They followed over 100 000 cell division events in up to 10 generations under normal conditions as well as heat stress.

Luo et al. (2008) designed a device with triangular- and later rectangular-shaped (Luo et al. 2009) cell chambers emanating from the main channel. The yeast cells can, thus be perfused with medium without being flushed away. As a proof-of-concept, they observed changing expression levels of the pCUP1 promotor in response to different Cu^2+^ concentrations. Other possibilities to isolate the cells in the cell chambers also exist, i.e. by manually closing the chambers after loading with valves (Park et al. 2013) or membranes (Charvin et al. 2008).

By implementing silicon oxide instead of PDMS as the material of choice in device manufacture, Huang et al. (2009) employed a roll-up technique to produce microchannels with a perfect circular cross-section and adjustable dimensions. The multistep manufacturing process involved forming a multilayered substrate using spin-coating, chemical vapour deposition, and chemical etching techniques, which creates an intrinsically strained SiO/SiO_2_ bilayer system that automatically rolls up upon release. Interestingly, yeast cells growing within the microchannel were observed to form a peculiar elongation in proof-of-principle experiments, an adaption to the constriction that was not, however, lethal to the cells.

#### Pressure-based active trapping

In contrast, some groups followed a different approach to obtain arrays of locally fixed cells. They devised a cell trapping method based on the flexibility of the elastomeric polymer, PDMS. Upon subjection to pressure the PDMS channels can be widened, allowing cells to enter, and upon relieving the pressure the PDMS returns back to its prior state, thus trapping cells within the channel. The schematic trapping principle along with a microscopic image of yeast cells trapped under elastic micropads is shown in Fig. 3(B).

Being the first to try this method, Groisman et al. (2005) probed the dynamics of large numbers of yeast cells at the microcolony level. Using a microfabricated chemostat, they were able to align bacteria, as well as yeast cells, in a biofilm-like manner using microchambers interspaced by hanging channel walls, leaving gaps of ∼0.6 µm between the wall and device bottom in the absence of pressure. These gaps permit exchange of substances, such as nutrients and waste, yet are impassable, however, to the cells. When a high gauge pressure (8 psi) is applied, cells can pass freely between the chambers. Thus, the cells can initially be distributed equally within the device, after which they can be isolated from one another. With this chemostat, colony growth behaviour and gene expression linked to quorum sensing were investigated; as such, dynamic response experiments were conducted and colonies subjected to temporarily changing exogenous stimuli. By addition of 10 nM of the autoinducer N-3-oxo-hexanoyl homoserine lactone to an *Escherichia coli* mutant with GFP(LVA) (tagged at C-terminal with amino acid sequence RPAANDENYALVA for faster degradation; Andersen et al. 1998) fused to the *luxR* gene, which is involved in quorum sensing, a rapid response (i.e. < 1 h) with an exponential increase in the fluorescence level of about 3–4 orders of magnitude was triggered. Upon removal of the autoinducer, this was followed by an equally fast decline in the fluorescence intensity, due to a rapid degradation of the GFP(LVA). While the main part of the investigations was conducted with bacterial cells, the group emphasized the potential of this or similar platforms for use with the yeast cells. They further highlighted the potential applicability of their device design for other studies, e.g. regarding aspects of gene expression and mutation, but also cell ageing, population dynamics, and sporulation (Groisman et al. 2005).

Lee et al. (2008) designed a device where whole channels are expanded and retracted upon the application and removal of applied pressure, respectively. In doing so, the yeast cells become trapped and are clamped between the channel ceiling and bottom. This was coupled with a sophisticated microfluidic circuit including different fluidic resistances, which facilitates a precise fluid and pressure control. Thus, flow velocity and pressure are decoupled, allowing key processes (i.e. flushing out nontrapped cells and nutrition) to be achieved without releasing trapped cells. This work was mainly concerned with creating an easy-to-use and easy-to-set-up system for widespread applications in cell biology. As a proof-of-concept, they monitored the expression of enhanced GFP (EGFP) from a Fus1 promoter region, which was activated by pulses of the α-factor pheromone intermitted by a washing step. The fluorescence signals, and hence cellular response was found to vary significantly from cell to cell, which was attributed, besides stochastic noise, to differences in the transcription and translation apparatuses of each cell. Possible effects deriving from different stages in the cell cycle were ruled out because all cells were pretreated with 200 mM hydroxyurea, arresting all cells in the S-phase. Similar set ups were later applied to investigations on stress response involving the high-osmolarity glycerol (HOG) pathway and crosstalk between the different signalling components (Lee et al. 2020, Uhlendorf et al. 2012).

Besides these studies, another big area of interest in yeast research is concerned with cell ageing and replicative lifespan. Therefore, obtaining isolated mother cells is desirable to study them in an unobstructed manner. Conventional methods involve manually removing budding daughter cells with a needle, a time-consuming and tedious task, which is also stressful for the cells because of the sheer size of the needle compared to the cell, making a precise dissection extremely difficult. Using microfluidic technologies, mother yeast cells can be trapped underneath micropads or pensile columns, while daughter cells that bud off are flushed away by a constant flow of medium. As an estimate, the dissection task that four to five trained people would achieve in 3–4 weeks, can be conducted with one microfluidic device in only 2–3 days (Zhang et al. 2012, Zou et al. 2017). As an additional benefit, nutrient medium is constantly refreshing in the microfluidic device. Furthermore, no agar, which lowers the resolution for microscopy, is needed. With such a device, Lee et al. (2012) discovered age-related morphological changes in cells of *S. cerevisiae*, with a so far unseen heterogeneity. By tagging VPH1, encoding a subunit of the vacuolar ATPase, with GFP, they focussed on the development and morphological changes in the vacuole. They found two cellular shapes at death, an ellipsoidal and a spherical shape. The shape correlated with the age of death, being 12 budding cycles for the spherical type and almost double the age for the ellipsoidal shape. The vacuole shape, herby, is either fragmented, fused, or tubular, the latter only occurring in ellipsoidal cells. Later, the group used the same device to investigate whether calorie restriction diets are indeed a reliable method to extend the live span of organisms (Huberts et al. 2014).

Differing from the other studies, Ohnuki et al. (2009) used a two-layered device design with a secondary air-filled channel through which pressure can be applied, trapping yeast cells in the main channel. Thus, they were able to trap the cells at will and on demand. Together with the semiautomatic image processing programme CalMorph (Ohtani et al. 2004), they were able to monitor cell cycle progression in *S. cerevisiae*. Another approach to trap yeast microcolonies was conducted by Frey et al. (2015), who manually pressed yeast cells down against PDMS clamping pads by bonding a glass cover slip onto the PDMS chamber after preloading with the cells using a pipette.

### Single-cell trapping

**Figure 4.**
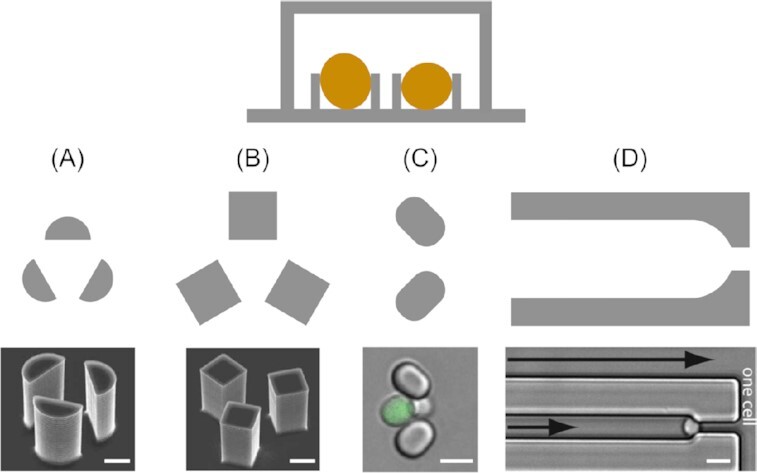
Overview of channel features used to trap single yeast cells. The above schematic depicts a cross-section taken through a microfluidic channel containing yeast traps, as well as top-down views of four different trap designs (A)–(D). Electron micrographs of semicircular (A) and square (B) three-pillar trap designs reproduced with modifications from Ryley and Pereira-Smith (2006) with permission from John Wiley and Sons (Licence Number: 5271491246941). (C) Microscopy image of an oval two-pillar trap design reproduced with modifications from Crane et al. (2014) with permission from the Creative Commons Attribution license (www.creativecommons.org/licenses/by/4.0/). (D) Microscopy image showing a channel-based trap design reproduced with modifications from Rowat et al. (2009) with permission from National Academy of Sciences. Scale bars represent 5 µm.

As described previously, a common challenge that most studies in the field of yeast research are concerned with is the trapping of single, individual yeast cells in a sustained manner, ensuring daughter cells that bud off are automatically removed from the mother cell. To meet this need, a suitable method concerns the design of physical flow traps; the trap dimensions should be the size of one mother yeast cell so as to accommodate only one single cell. Different shapes were used to design trapping structures within a microfluidic device. An overview is illustrated in Fig. 4.

Ryley and Pereira-Smith (2006) proposed three different trapping structures, dubbed ‘yeast jails’. A total of two of those, featuring groups of three micropillars per trap, were successfully used to capture yeast mother cells (Fig. 4A and B), while the third (a ‘horseshoe’-shaped trap) was disregarded on the grounds of less space efficiency compared to the other designs. One minor drawback of these early designs was that in order to effectively remove the daughter cells, constant reversal of the flow direction was necessary. A constant flow provokes pseudohyphal growth, which in turn leads to daughter cells remaining attached to the mother yeast cell.

With the two three-pillar systems, the authors then investigated RAS2 and HSP104 expression, genes known to affect survival rates and life span. It was found that the expression levels vary significantly from cell to cell, with differences in fluorescence signals ranging from up to 2-fold for RAS2 and up to 9.6-fold in the case of HSP104. These findings, impossible to detect with conventional nonmicrofluidic methods, suggest that the expression of RAS2 is a key factor in varying life spans in otherwise identical yeast cells. As for HSP104, which is known to play a major role in survival strategies against external stressors, it was shown that the ability to track single cells over time allowed factors influencing survival, e.g. duration, type of stress or HSP104 basal levels, to be thoroughly investigated.

As their system was simple and effective, it was later adapted in similar studies (Ryley and Pereira-Smith 2006). Liu et al. (2015) used the same three-pillar system to trap cells and implemented their device in an automated high-throughput platform for investigations on cellular lifespan and gene expression. It was dubbed HYAA (High-throughput Yeast Aging Analysis) and later developed further into the HYAAC (for Cryptococcus) to accommodate another yeast, *C. neoformans* (Orner et al. 2019). The virulence and antifungal resistance of these pathogenic fungi is dependent on ageing and age-related phenotypes. Studies on their lifecycle as well as ageing are, therefore, highly needed and important for effective treatment (see also the ‘Fungi-on-a-Chip technologies for medical applications’ section).

Crane et al. (2014) developed ‘*A Long-term Culturing And TRApping System*’, in short ALCATRAS, which diverged from the complex structures detailed in previous studies and exploited a very simple, yet efficient, array of cell traps. The two-pillar based system, as illustrated in Fig. 4(C), is able to catch flowing yeast mother cells, where a constant stream of fluid is required to keep them inside the traps and also flush away budding daughter cells. This straightforward approach offers a trapping efficiency of almost 100% and is easy and inexpensive to use and manufacture. Furthermore, the design allows for a high density of traps, and thus raises the level of data acquisition by an order of magnitude compared to similar devices. Moreover, the platform enables fast switching of media, as shown in a glucose limitation experiment. Upon monitoring the transcription factor Msn2p, which is involved in cellular stress response in *S. cerevisiae*, it was found that switching the medium to low glucose levels entailed an immediate relocalization of Msn2p-GFP to the nucleus, thus triggering the cellular stress response. This was followed by repeatedly switching between high (2%) and low (0.1%) glucose levels in intervals of a few hours. Interestingly, it was observed that after each cycle, fewer cells responded with nuclear localization of the Msn2p-GFP, indicating a learning behaviour. Since the stress was not persistent and a complete stress response became unnecessary, the cells might have reduced their stress response to save energy. Alternatively, they suggested that this observation could be caused by cell ageing. In a second experiment, Whi5p, a transcriptional repressor, expressed specifically in the transition from M to G1 phase, was used to gain new insights into the cell cycle of yeast cells. A periodic signal, representing cell division times, was measured, which was found to be strongly dependent on age and varied from individuum to individuum. These results were only made possible by the ability to precisely trap and track individual mother cells, while instantly and automatically removing budding daughter cells, demonstrating the usefulness of this type of microfluidic device. A very similar, two-pillar based design was later used by Jo et al. (2015) and for cell ageing studies and investigations on age-related disease (Jo et al. 2015, Jo and Qin 2016, Yu et al. 2020). Another group used similar arrays for studying the expression and inclusion of the Parkinson’s disease associated protein, *α*-synuclein (Fernandes et al. 2014, Rosa et al.^2012^).

Differing slightly to the aforementioned studies, Rowat et al. (2009) developed trapping channels, termed lineage chambers, instead of single traps, as illustrated in Fig. 4(D). They mainly trapped single cells at the channel ends following a Poisson distribution. As a result, all of the daughter cells that budded off remained within the channel, aligned in single file behind the mother yeast cell. Thus, using a GFP-tagged heat-shock protein Hsp12, they showed how such nonessential elements fluctuated randomly, but synchronized between mothers and daughters, while on the other hand essential proteins such as RPS8b-GFP expression is comparably constant throughout the colony and over time. A similar design, having shorter, dead-end channels, was designed by Fehrmann et al. (2013). They monitored shorter lineages for tracking mitochondrial dynamics involved in rejuvenation and cell ageing.

**Figure 5.**
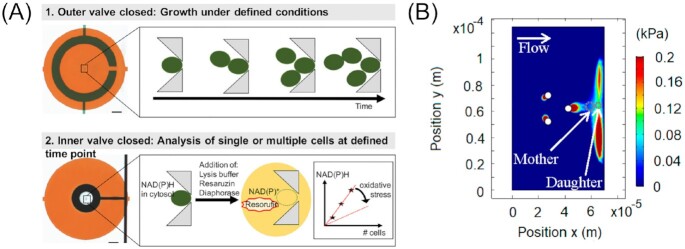
Single-cell trapping of yeast cells using on-chip valve and slipstream techniques. (A) The microfluidic trapping device allows a valve-controlled two mode operation, a dynamic growth study, as well as end point/time point analysis of lysed cells. Image reproduced with modifications from Stratz et al. (2018) with permission from John Wiley and Sons (Licence Number: 5271500367493). (B) A microdevice for contactless trapping utilizing the slipstream effect. Image reproduced with modifications from Duran et al. (2020) with permission from the Creative Commons Attribution license (www.creativecommons.org/licenses/by/4.0/).

Stratz et al. (2018) designed a multifunctional microdevice, which combines both single-cell studies and studies on cell culture level under defined conditions. Their sophisticated two-valve system facilitates the isolation of cells, featuring a trapping system based on two triangular pillars that point towards one another. By switching the valves, it is possible to define two states around the trapped cells, as illustrated in Fig. 5(A). When the outer valve is closed, the yeast cells remain in contact with the surrounding medium, allowing for diffusion-controlled nutrient supply, removal of cell excretes and protection from shear stress induced by flowing medium. Upon closing the inner valve, the cells are entirely cut off from the surrounding environment. As a proof-of-concept, the authors investigated intracellular NAD(P)H levels in *S. cerevisiae* single cells and its dependency on stress factors. Therefore, the enzyme diaphorase and the substrate resazurin were added to the buffer and the trapped single cells were lysed. The released NAD(P)H then functions to reduce resazurin to the pink-coloured and highly fluorescent resorufin. Due to the small analysis volume in the secluded cell chambers, dilution effects are strongly minimized, which enables very precise measurements of metabolites ranging down to attomoles. It was found that the NAD(P)H levels in yeast mother cells are mostly unaffected by a stress treatment with hydrogen peroxide, although this varied strongly from cell to cell. With these precise investigations, it was shown that this platform is well-suited for further bioanalyte studies on-chip.

The main aspect concerning each of the previously explained trapping systems relates to the physical nature of the method. It was commonly assumed that the mechanical contact with the traps had no or negligible impact on the cells (Wu et al. 2004). Duran et al. (2020), however, reinvestigated the matter and found a pressure dependent deformation of the cells in the range of a few submicrons, as well as a slight decrease in expression levels of a representative constitutive gene at the usual pressures exerted on the cells in physical traps. This suggests that trapping systems that involve physically ‘grabbing’ the cells may have an impact on the yeast cells, possibly altering outcomes of the investigations. Hence, the ‘Mother Machine’ device developed by the group omits any physical wall contact with the individually trapped yeast cells by exploiting the so-called slipstream effect (Fig. 5B). This sophisticated, yet simple, design is based on an array consisting of groups of three micropillars, which creates a disturbance in the flow, i.e. by inducing a pressure hole behind the pillars equivalent to the size of a yeast mother cell. While these are tightly entrapped, budding daughter cells are simply washed away. Importantly, the exerted pressure on the yeast mother cells in this wall-free environment is on the order of a few hundred pascals, as opposed to hundreds of kilopascals in the conventional devices, yet maintains a sufficiently firm grip around the cells.

Most of the above-described methods are immensely beneficial and impactful, however, tend to be quite elaborate and technically challenging, often requiring both specific expertise as well as very specific and often expensive equipment. In order to make these techniques accessible for a broader range of scientists and institutes, Cabrera et al. (2017) set out to create an easy-to-use device, which combines a simple, commercially available microfluidic device with a genetic tool to repress the formation of daughter cells. Chen et al. (2019) identified imaging as the most problematic and inaccessible aspect of these studies. Therefore, they designed simplistic and affordable hard- and software add-ons for automated microscopy of microfluidic single-cell study devices, by modifying existing imaging equipment.

#### Cell separation techniques

As yeast cultures commonly consist of not just single cells, but also budded doublets and clusters, trapping of single mother cells for the described studies is not a trivial task. Hence, automated or even integrated microfluidic devices to facilitate separation procedures are highly desirable. Cell collection and separation is a common and important task in many biological applications, e.g. for the removal of pathogens (see also the ‘Fungi-on-a-Chip technologies for medical applications’ section), as an experimental preparation step or to select certain high-performing entities from low-performing ones. Harnessing the benefits of microfluidics, numerous methods have been developed and applied with fungal spores (Park et al. 2019, Wang et al. 2020) as well as other nonfungal cell types, such as cancer cells or bacteria (Lee et al. 2013, Tang et al. 2019, Xiang et al. 2019, Yan et al. 2017). The yeast-focussed platform by Liu et al. (2021), which uses the viscoelastic liquid polyethylene oxide (PEO) as a sheath fluid to separate different shapes of *S. cerevisiae*, is another example of such a technique. By injecting a stream of PEO into the cell suspension and pumping through a rectangular microchannel, both elastic and inertial lift forces are exerted onto the yeast cells. This enables a separation into singlets, budded doublets, and clusters, based on shape and size. While doublets were still found in all of the outlets, it was possible to remove all clusters from the singlet fraction, as well as enrich the proportion of clusters through a different outlet by a factor of two. In this study, which was purely focussed on developing a separation device for *S. cerevisiae*, the effects of channel dimensions, polymer concentration, and flow rates of both sample flow and sheath flow on the separation success were analyzed. By conducting the SYTO 9 and EthD-III cell viability tests, harmful stress on the cells caused by the separation could be ruled out. The final, optimized microdevice opens new avenues for research or practical applications in industry or medicine, where only one or two of the different shape-types of *S. cerevisiae* is the desired subject.

### Droplet-based microfluidics for applications in yeast biotechnology

**Figure 6.**
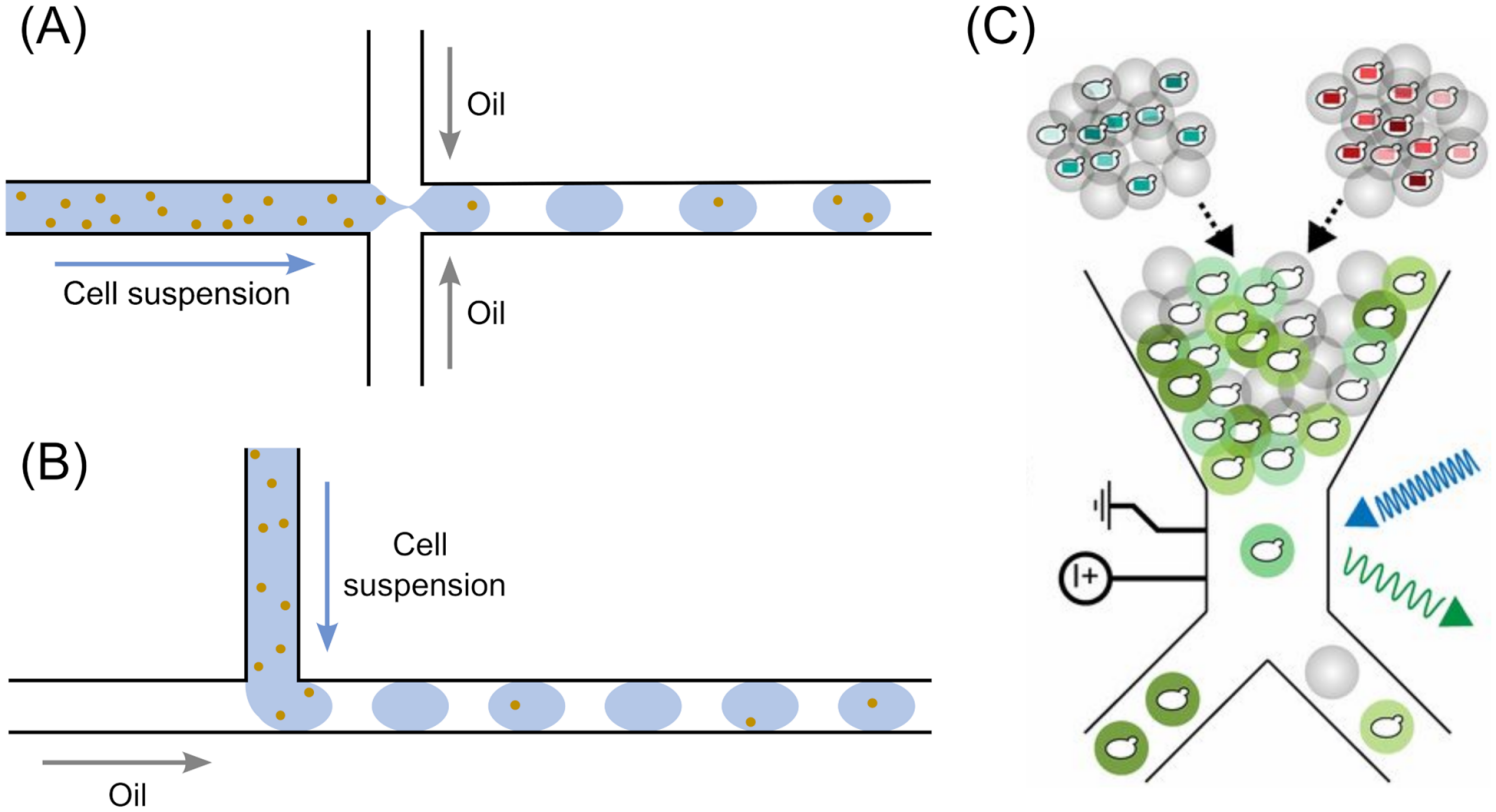
Droplet-based microfluidic methods. Schematics illustrating droplet generation using (A) flow focussing or (B) T-junction techniques. (C) Image showing the principle of fluorescence-activated droplet sorting as described in Wang et al. (2019), which was used to sort yeast genotypes with high amylase activity from a randomized library. Image reproduced with modifications from Wang et al. (2019) with permission from the National Academy of Sciences.

Another very important trait of the universal model organism and working horse, *S. cerevisiae*, as well as other yeasts, is their capability to be easily recombinantly modified, either for fundamental research or for biotechnological applications. Already a yeast’s natural capacity to produce bulk chemicals, like ethanol (Basso et al. 2011), isopropanol (Nandy and Srivastava 2018), or citric acid (Cavallo et al. 2017) as well as enzymes (Bussamara et al. 2010, Zaky et al. 2014) and pharmaceuticals (Walsh 2014) is huge. Facing the ongoing irreversible global deprivation of fossil resources, mainly oil and coal, as a source for basic chemicals, the responsibility of the biotechnology industry to provide these resources in the future will continue to grow. With similar severity, medicine has been confronted with a constant increase in antibiotic resistance. In the last decades, this process was further accelerated by industrial livestock farming with its mass use of antibiotics. Therefore, high through-put screening (HTS) for new yeast strains producing new substances, with higher yield is essential. Microfluidic platforms have proven to be an ideal tool for this, especially droplet-based methods (Box 2, Fig. 6), where thousands of parallel reaction vessels can be generated. So far, while being an extremely young field for yeasts, it has already been successfully applied to other organisms, such as mammalian cells, bacteria, or whole microbiota (Balasubramanian et al. 2021, Brouzes et al. 2009, Trivedi et al. 2010, Tu et al. 2021, Watterson et al. 2020).

Box 2. **Droplet-based microfluidics**

Droplet-based (or segmented flow-based) microfluidic technologies form a major subdiscipline within the field of microfluidics (Griffiths and Tawfik 2003, Tawfik and Griffiths 1998). Typically, two immiscible fluids are dispensed into a microchannel employing either the T-junction or flow focussing technique, resulting in the formation of microdroplets contained within the carrier liquid, as illustrated in Fig. 6(A) and (B). Commonly used carrier fluids include perfluorinated alkanes, such as perfluoromethyldecaline. The secondary phase is usually an aqueous solution or suspension, which can also be a solidified hydrogel. Aqueous droplets dispersed in an oil carrier fluid are known as ‘water-in-oil’ microdroplets; conversely, oil droplets dispersed in an aqueous carrier fluid are known as ‘oil-in-water’ microdroplets. If generating ‘water-in-oil’ microdroplets, for example, a hydrophobic channel material will aid droplet formation. Indeed, hundreds to thousands of separate reaction or incubation vessels can be created per second, depending on the degree of parallelization within the device (Baret 2012). With a static incubation, mixing within the droplet only occurs through diffusion. An improved mixing can be achieved through chaotic advection, instigated by incorporating meandering microchannels with narrow loops into the device design. The oscillating shear stress invokes a turbulent flow within the droplets, leading to thorough mixing within milliseconds (Bringer et al. 2004).

The carrier liquid, into which the microdroplets are dispersed, prevents cross-contamination. It also typically contains a surfactant that sits at the oil–water interface to stabilize droplet formation. This is especially important if the droplets are kept in a loose and random array, without physical interspacing, after initial droplet generation (emulsion-based microfluidics; Bauer et al. 2010, Bremond and Bibette 2012). Such amphiphilic additives, however, might intervene with the chemical reaction studied or can be toxic to some organisms, though biocompatible surfactants are being developed (Baret 2012, Wagner et al. 2016). The microfluidic droplet ‘toolbox’ allows various microdroplet unit operations to be performed, including droplet merging, splitting, and synchronization, as well as targeted injection of material into existing droplets (dosing; Berry et al. 2019, Köhler and Cahill 2013, Pekin et al. 2011) and integrated fluorescence-activated droplet sorting (FADS; Fig. 6C; Baret et al. 2009). Overall, droplet-based microfluidics afford significantly increased experimental throughputs and reduced reagent consumption compared to conventional methods, as well as automated pipetting robots. Moreover, high throughput experimentation leads to increased precision of the measurement with more datapoints collected (Miller et al. 2012).

A droplet-based microfluidic approach was employed by Hosokawa et al. (2015) for screening a metagenomic library for microbial enzymes. They extracted a fosmid library (Kim et al. 1992) from a soil microbe population and transfected the F-plasmids into *E. coli* and *S. cerevisiae*. With their microfluidic droplet generator, they encapsulated the transfected cells plus fluorescein dicaprylate as a fluorogenic lipolysis reporter into ∼100 pl droplets. The droplets were stabilized with a surfactant and subsequently solidified with agarose to further improve droplet stability and thus segregation of the microenvironments. When a lipolytic enzyme is expressed within the capsule, the hydrolysis of fluorescein dicaprylate is catalysed and thus triggers the generation of a fluorescence signal. Fluorescent gel microdroplets were then sorted using fluorescence-activated cell sorting (FACS), isolated on plate and eventually characterized as well as sequenced. Using this method, they were able to screen 67 000 clones in 24 h with an overall reagent consumption of less than 10 µl. As a result, the esterase EstT1 was discovered and isolated. Later, the group used the platform to screen for ligands against G-protein coupled receptors (GPCRs), which are involved in many pathogenic signalling pathways of various diseases, for drug discovery. Resulting from their screening cycle, they discovered new functional ligands, occasionally with even higher activity than exendin-4 (Ex4), a well-known ligand. As a future improvement for the screening system, they proposed running several subsequential cycles to enhance overall resolution, e.g. by excluding nonspecific yeast cells from droplets with more than one cell (Yaginuma et al. 2019).

A further addition to the enormous portfolio of applications for this immensely beneficial high-throughput screening technology was made by Wang et al. (2019), with their droplet-based study on RNA interference processes. The improvement of protein secretion of an organism with RNA interference is, despite appearing counterintuitive or even paradoxical at first, a feasible technique. However, it has been impossible to employ this method in a targeted and planned manner to date, due to a deep lack of understanding regarding the intracellular mechanisms taking place at all stages of the process, i.e. synthesis, post-translational modifications, and secretion of the protein, involving transcriptional factors, promoters or inhibitors, protein transporters, and beyond. Therefore, an immensely high-throughput method is needed to screen randomized libraries for expression-enhancing RNAi candidates, which in turn would further accelerate the elucidation of functional mechanisms underlying this phenomenon. The group used a similar set up as featured in the above discussed studies, with an integrated FADS for sorting (Fig. 6C). In their proof-of-concept model system, they used α-amylase expressed in *S. cerevisiae*, combined with boron–dipyrromethene–starch as a fluorogenic substrate. Using this set up, they managed to narrow down the initial 1 million cells to a sample of 340 cells with potentially enhanced amylase production, in only three subsequential cycles, with ∼7 h for each in-droplet assay cycle. Thus, the number of potential candidates was reduced to a sensible degree for further, more detailed and elaborate analyses. As a result, several novel amylases with a maximal improvement of 2.2-fold were recombined. Furthermore, it was found that genes associated with various cellular processes, such as metabolism, protein modification, or degradation and cell cycle, are involved in the modification of protein production. Moreover, they proved the applicability of their system to further studies on RNAi protein secretion modifications and mechanisms.

Another technique that immensely benefits from the droplet-based microfluidics approach is the so-called isogenic colony sequencing (ICO-seq). The problem with using mRNA for sequencing, is that mRNA concentrations are inherently very low. By encapsulating single cells from a randomized library into hydrogel droplets, the cells grow into isogenic colonies, thus automatically amplifying the mRNA for subsequent deep screening. Because the RNA stems from only one initial cell, a direct correlation between genotype and phenotype can be drawn. Liu et al. (2019) applied this technique to perform a heterogeneity analysis of a *S. cerevisiae* ARO4 mutagenesis library, as well as to analyze the underlying mechanisms for switching between white and opaque cells in *C. albicans*.

Besides these two yeasts, droplet-based microfluidics has also been employed with *Yarrowia lipolytica* (Beneyton and Rossignol 2021). This dimorphic yeast has enormous potential for the production of heterologous proteins combined with a high secretion power and has very recently become subject of further microfluidic investigation with an interest for biotechnological applications in the fields of biofuels and bioremediation (Lesage et al. 2021).

Not only yeasts, but fungi in general, due to their role as decomposers in nature, have an enormous repertoire of exudates and metabolites, including numerous enzymes and small molecules, useful for biotechnological applications. Thus, extending microfluidic high-throughput screening onto a wider range of fungi, in particular filamentous fungi, is highly desirable. So far, filamentous fungi featured in microfluidic HTS studies have been *Aspergillus nidulans* (Beneyton et al. 2016) and *Trichoderma reesei* (He et al. 2019), leaving much potential yet for applications in the future.

## Fungi-on-a-Chip technologies for medical applications

Another important aspect of many fungi is their pathogenicity. One of the most common causes of nosocomial disease is the fungus *C. albicans*. In its yeast form, dimorphic *C. albicans* becomes quickly disseminated. Once in the body, however, it switches to its filamentous form and is, thus, able to penetrate host tissue and cells. Alongside other *Candida* species, the opportunistic pathogen is known for causing candidiasis in immunocompromised patients, an illness, which once acquired, proves fatal in about 40%–60% of the cases (Uppuluri et al. 2017). Even though the severity of this fungal threat has been known for decades, the frequency of cases is not expected to diminish in the future (Pfaller and Castanheira 2016). With all of these fungal diseases, the most crucial aspect is the time to diagnosis (and treatment), often a key factor determining the life or death of the patient. It has been approximated that antifungal intervention should be started no later than 6 h after the onset of symptoms for an effective treatment with the highest chances of a full recovery and fewest complications (Fuchs et al. 2019).

Conventional methods for diagnostics are slow and usually comprise initial blood and plate culture, followed by polymerase chain reaction (PCR) analyses. Therefore, new and much faster techniques for detecting, and possibly even removing, blood-borne pathogens from the patient’s blood need to be developed. Faster identification of the prevalent pathogen would further prevent inappropriate initial antifungal treatment, which in turn again harms the patient and facilitates the development of drug-resistance (Morrell et al. 2005, Zhang et al. 2020). Besides this direct clinical relevance, *C. albicans* is also a well-known model organism for studies of fungal biology. Microfluidic technology allows for rapid and precise processing and detection on the microscale. Furthermore, laminar flow profiles can be exploited to allow membrane-free dialysis systems to be designed. These attributes are desirable for the development of diagnostic and therapeutic devices for those diseases involving microbes.

### Microfluidic techniques for filtering pathogens from blood

**Figure 7.**
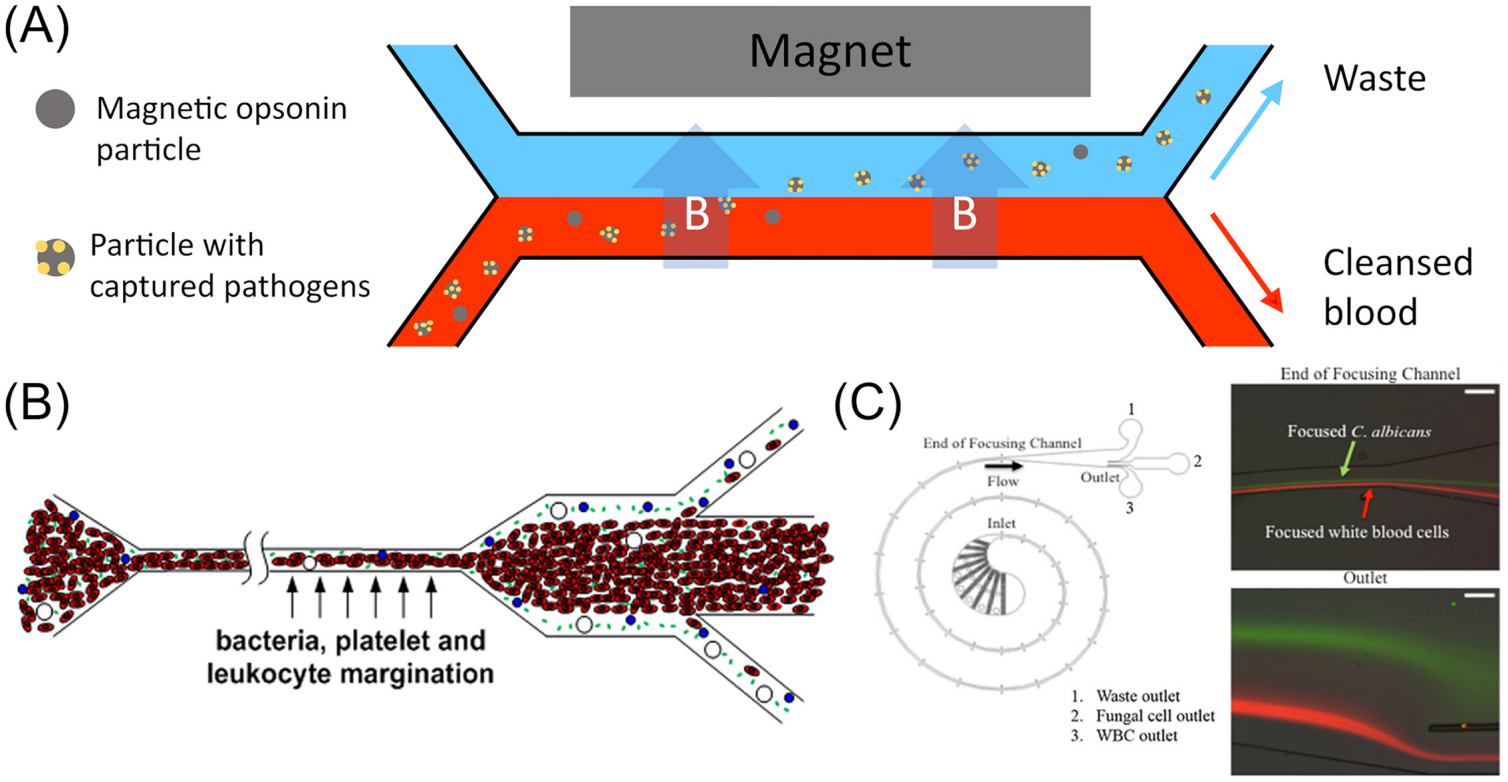
Microfluidic devices for blood cleansing tasks. (A) A dialysis-like microfluidic device employed to filter fungal pathogens from blood via magnetic opsonins beads specific to the targeted pathogen. The particles with the attached pathogen are then extracted from the blood into an aqueous parallel waste collection stream using a magnet. The illustration is based on Yung et al. (2009). (B) Microdevice used to filter pathogens from blood relying on margination. As observed in blood vessels, red blood cells tend to migrate to the channel centre resulting in other blood components such as platelets or microbes being pushed towards the channel walls. Subsequently, they can be removed by a sudden widening of the microchannel. Image reproduced with modifications from Hou et al. (2012) with permission from AIP Publishing (Licence Number: 5271501114499). (C) A device, termed the Inertial Fungal Focuser (IFF), used to remove fungal pathogens from blood via inertial focussing. Image reproduced with modifications from Fuchs et al. (2019) with permission from the Creative Commons Attribution license (www.creativecommons.org/licenses/by/4.0/). Scale bars represent ca. 100 µm.

In an attempt to develop a clinical blood filtration device to tackle candidiasis, Yung et al. (2009) employed a microfabricated high-gradient magnetic field concentrator (HGMC) for *E. coli* (Xia et al. 2006) as a prototype for redevelopment as a microfluidic platform for *C. albicans*. The basic idea involved coating superparamagnetic microbeads with pathogen specific antibodies. The bead suspension was then pumped parallel to the blood sample through a microfluidic separation channel, similar to a dialysis machine, but without the need for a physical membrane (i.e. as the device operates under a laminar flow regime). At the interface, the magnetic opsonins then pair with the pathogen and can be extracted from the blood, as illustrated in Fig. 7(A). By optimizing the channel design and arrangement, as well as using a tuneable electromagnet, they were able to optimize the device’s efficiency to achieve a cleansing rate of 80% in human whole blood using a volumetric flow rate of 20 ml/h. While both rates would not be sufficient to treat a patient, therapeutically relevant standards can be achieved by combining several devices in multiplexed arrays, thus enhancing the total throughput and final cleansing rate.

To further develop a label free alternative, Hou et al. (2012) utilized the naturally occurring microcirculatory phenomenon of blood cell margination, thus avoiding the need for magnetic beads. In microtubes (e.g. blood vessels), red blood cells tend to migrate towards the centre of the channel due to their shape and deformability, while other particles (e.g. white blood cells, platelets, and bacteria) tend to be pushed towards the channel walls. A sudden expansion of the channel then allows bacteria, as well as inflammatory cellular components (e.g. platelets and leukocytes), to be extracted through side outlets, as illustrated in Fig. 7(B). In a two-stage design, this step is repeated, resulting in a cleansing rate in whole blood of 90% for *S. cerevisiae* (model organisms simulating pathogenic fungal cells such as *C. albicans*) and > 80% for sepsis-related blood components. Using this filtering principle, a simple and continuous dialysis setup was manufactured, which does not require any sample preparation and enables the return of the filtered blood back to the patient. The only drawback is the relatively low throughput of 1 ml/h, compared to the microbead-based methods, which again could possibly be enhanced by numbering-up.

Again, utilizing the magnetic particle principle, Kang et al. (2014) put emphasis on improving the used opsonin-bead entity. By engineering a mannose-binding lectin (MBL) complex bound to the human immunoglobin IgG1 Fc, which functions as a linker to the superparamagnetic nanobeads, they were able to significantly broaden the range of targeted pathogens. As part of the innate immune system, MBL detects and binds certain carbohydrate structures exhibited on the surface of human pathogens, i.e. fungi, bacteria, viruses, as well as protozoa (Tomaiuolo et al. 2012). Harnessing this ability is highly beneficial to tackle unspecific sepsis without needing to identify the pathogen first, thus saving time crucial for the survival of the patient. Further, this allows to fight infections with common nosocomial pathogens, such as MRSA, which are unresponsive to conventional treatments. In terms of channel design, the authors mimicked the vascular channel design of the venous sinusoids of a human spleen, hence the device’s name ‘biospleen’. A total of two microchannels, interspaced by rectangular slits, allow filtration with different flow velocities in the channels, even with stop-flow regimes, meaning that one fluid rests while another one is streaming past it. Another important aspect they improved concerns the extraction of the opsonin particles from the blood sample. Due to the bead diameter of 128 nm, their magnetic moment is relatively small, resulting in a removal efficiency of only 80%. By adding larger, uncoated 1 µm diameter beads, the local magnetic fields were enhanced, resulting in the smaller opsonin beads being dragged along and, therefore, enhancing the removal rate to > 99%.

While these devices are very well-suited to the removal of unspecific pathogens from blood, it is unfit for microscopical detection and diagnosis of the pathogen. Therefore, the design was refined by Cooper et al. (2014) to incorporate an array of microwells in the channel ceiling, having a diameter of 50 µm and a height of 20 µm, together with a ferromagnetic flux concentrator between the magnet and the channel to create a uniform magnetic gradient along the channel. Thus, they were able to capture pathogen-bead conjugates in an evenly distributed manner to facilitate imaging, without the beads obstructing one another. Using this set-up, approximately 70% of the initially seeded, fluorescently labelled *C. albicans* were detected, both in saline and whole blood (operated using a volumetric flow rate of 10 ml/h). That translates to a sensitivity of one fungal cell per ml in less than 3 h. The ability to concentrate diluted pathogens from blood samples and subsequently remove them from the channel is predicted to accelerate culture-based antibiotic susceptibility tests by a further 24 h, again crucial for a successful treatment of sepsis.

This represents a clear improvement compared to the device developed by Javanmard et al. (2012), where only 6.7% of the introduced *C. albicans* could be detected. Their channels are coated with anti-*C. albicans* antibodies, where the capturing mechanism is purely coincidental and relies solely on diffusion. The previously described set-ups utilize magnetic nanobeads to actively ‘forage’ for the pathogens. A similar microfluidic immunoassay was later developed by Asghar et al. (2019) to detect *C. albicans* from whole and lysed blood. In comparison, they achieved capture efficiencies of between 60% and 80% in run-times of less than 2 h.

Another attempt to circumvent the use of magnetic beads was conducted by Fuchs et al. (2019). Since the opsonin beads needed to be applied with a 1000-fold excess, eliminating these from the filtration principle would reduce costs significantly. Moreover, they designed a device, i.e. fully independent from any kind of labelling, called the Inertial Fungal Focuser (IFF; Fig. 7C). Their separation method relies on the phenomenon of inertial focussing. Due to the channel dimensions and parabolic flow profile, a wall force, directed away from the channel wall, and a shear force, directed towards the channel wall, are exerted on particles carried within the streaming fluid. The opposing effects result in an equilibrium position of the particle, which is strongly dependent on size and shape of the entity. A third force, called the Dean force, was introduced by incorporating a curved section into the channel design to improve the equilibration and specificity of the particle separation. With a recovery rate of 8.4% for different *Candida* spp. from lysed blood, they were able to trump the rate achieved by Javanmard et al. (2012) slightly, although still lagging significantly behind the aforementioned bead-based principles. Since the inertial flow effects are strongly dependent upon the viscosity of the liquid, these methods struggle in their handling of full blood samples, resulting in a relatively low filtration fidelity, even with lysed blood. Hence, the IFF device is not fit for blood cleansing tasks, however, it is applicable to concentrate and isolate fungal pathogens from blood, without the need for elaborate preparation steps. Thus, enough fungal material can be acquired within only 125 min using a volumetric flow rate of 24 ml/h to perform subsequent analyses downstream, such as PCR. Conventional methods, in contrast, take up to 5 days (plate-based) or 12–48 h (automated method), respectively. As well as enhancing the detection speed, the IFF significantly decreased the lower concentration limit for detection, with only 1600 cells/ml used in the experiment.

Besides these methods, another technique applied to sort cells using microfluidic set ups involves the use of electrodes. Such an approach was used by Cheng et al. (2007) to remove *C. albicans* from mixed cell cultures.

Very recently, many of the hitherto featured microfluidic disciplines and techniques were finally integrated into an automated diagnostics system, which was already launched in the field of medicine. The ePlex digital microfluidics platform by GenMark Dx combines on-chip PCR and DNA-hybridization-based probing with electrowetting (Choi et al. 2012) as the means to control the sample transportation and processing to detect fungal pathogens from whole blood samples (GenMark Dx 2021). Zhang et al. (2020) conducted a large-scale clinical evaluation of the system with real-live samples from several clinics as well as contrived samples containing clinically relevant fungal pathogens like *Candida* spp*., Cryptococcus* spp*., Fusarium* spp., as well as *Rhodotorula* spp. and compared the sensitivity, specificity, and overall performance with conventional and established methods such as bidirectional PCR, peptide nucleic acid fluorescence *in situ* hybridization (PNA-FISH), and matrix-assisted laser desorption ionization-time of flight mass spectrometry (MALDI-TOF MS). Strikingly, they found that neither the sensitivity nor specificity ever fell below 96% for any of the 15 species tested. In some samples, the automated platform was even able to detect pathogens, which were missed with the standard procedure. The time needed for a single run, which can be conducted with several samples in parallel, is approximately 100 min and, thus much faster than the above-mentioned conventional methods.

### Microfluidic drug-screening methods

After diagnosis and identification of the pathogen, targeted drug-treatment needs to be deployed. Here too, microfluidic technologies can be highly beneficial for the development of antifungal drugs. With an enormous throughput and significantly reduced reagent consumption, microfluidic approaches, such as droplet-based microdroplet technologies (as discussed previously in the ‘Droplet-based microfluidics for applications in yeast biotechnology’ section) can also be used for testing the efficiency of drugs against fungi. In a first proof-of-concept study using this approach, Zheng et al. (2017) screened four antifungal drugs (5-fluorocytosine, amphotericin B, terbinafine, and caspofungin) against *C. albicans*. Their examination, performed using the microfluidic device and Alma blue as fluorescent dye, took only 2 h and was found to be in good agreement with the microwell plate-based result. Using a similar, but microscopy-assisted approach, Qiang et al. (2019) developed a platform that combined rapid throughput techniques with precise imaging. Thus, they reported an 18-fold reduction in the time needed to screen drug libraries, compared to conventional well-plate methods, with 50 520 drugs screened in only 1 week. Moreover, their analysis technique was even more precise; as the fungal cells are aligned one after another in their respective microchannels, the fluorescently labelled cells can be counted and checked for vitality directly, while conventional methods rely on indirect photometric analysis. Additionally, the volumina of the tested substances required are 100 times lower using the microfluidic chip, thus saving money and resources.

### Microfluidic approaches for the development of antifungal strategies

Another way to fight these types of diseases is to prevent infection altogether. Understanding infection mechanisms and pathways aids the development of novel antifungal strategies. Again, microfluidic platforms offer a great potential for studies on these processes mainly taking place on the microscale.

Exemplary to that, Richter et al. (2007) set out to investigate *C. albicans* and *Pichia pastoris* biofilms, a key factor in the spread of nosocomial fungemia. Therefore, they developed a microfluidic platform with integrated dielectric microsensors. This set up allows for a contact-less measurement of biofilms and morphological changes triggered by external stimuli in real-time. Both the effects of mechanical shear stress as well as the drug response against the antifungal agent Amphotericin B was, thus monitored and revealed significant changes in growth dynamics. The fungicide caused a stress-induced arrest of replication, while on the other hand repair mechanisms were up-regulated.

**Figure 8.**
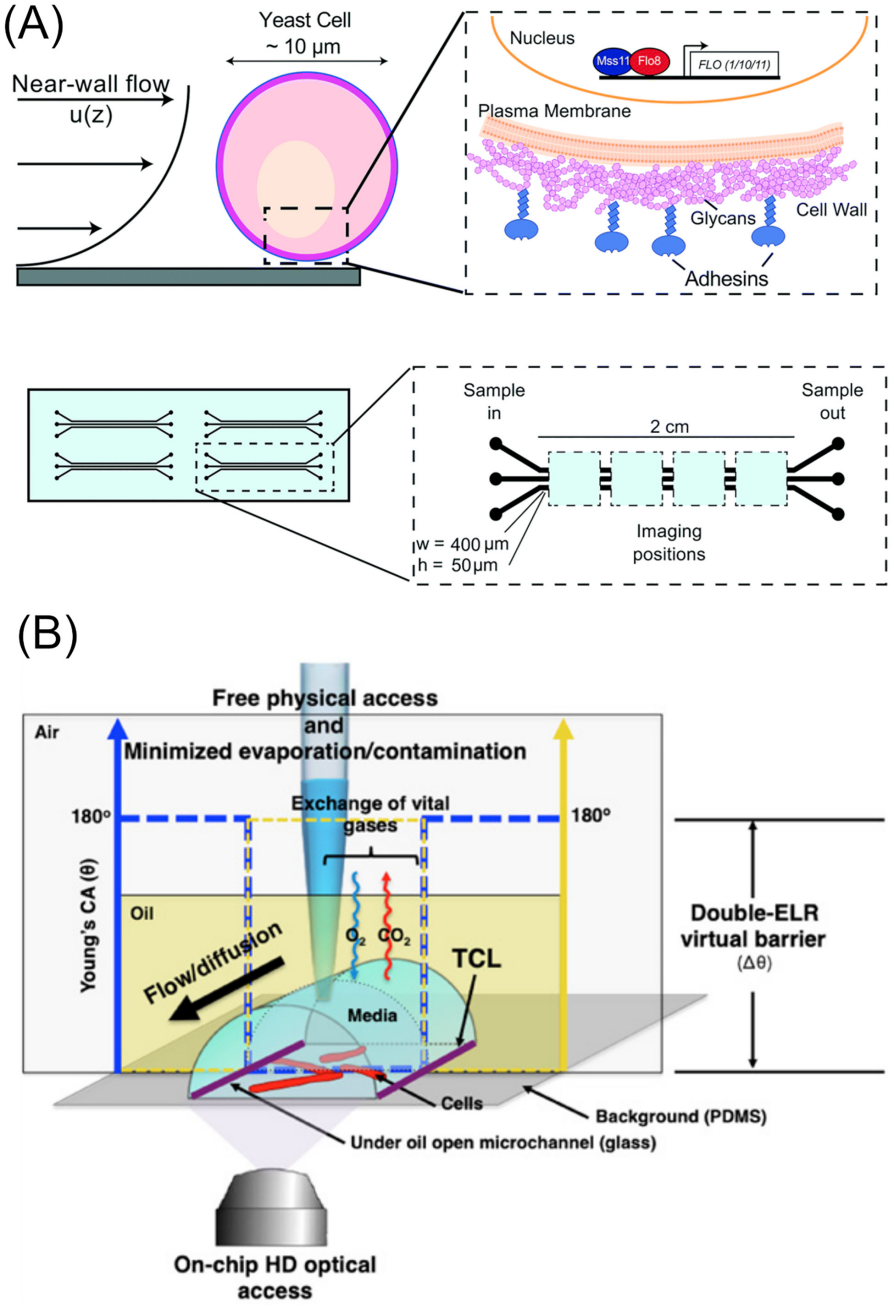
Microfluidic devices to study preinfection aspects of fungal pathogens. (A) Microdevice used to analyze surface adhesion of pathogenic yeasts under different shear-stress conditions. Shown are the principles of cell adhesion in yeasts, involving adhesin proteins under sheer stress (top) and the device design featuring arrays of parallel microchambers (bottom). (B) Under-oil microfluidic device for studying pathogenic fungal biofilm dynamics. The three-phase system consists of the PDMS substrate with micropatterned glass channels, an oil layer covering the channels and the aqueous medium or cell suspension, which is commonly injected into the oil phase. Images reproduced with modifications from Li et al. (2020) and Reinmets et al. (2019), respectively, with permission from the Creative Commons Attribution license (www.creativecommons.org/licenses/by/4.0/).

Similarly, Reinmets et al. (2019) used microchannels fabricated from either PDMS (usually hydrophobic) or glass (hydrophilic) substrates to investigate cell-surface as well as cell–cell adhesion in yeasts, key elements in biofilm formation (see Fig. 8A). Using the model organism *S. cerevisiae* it was found that the adhesive strength is highly dependent upon the genetic background of the fungi and differs significantly between strains. Beyond the medical implications, they further propose that this technology is able to aid studies in protein engineering, where adhesive forces of engineered yeast cells displaying surface proteins can be translated into affinity measurements. From a more practical angle, exploitation of this phenomenon also offers the opportunity to develop novel tools for cell separation based on different adhesive forces.

The dispersion of fungi from established biofilms marks the next step in the infection pathway. Li et al. (2020) approached this topic, introducing and utilizing a very new methodology in the field of microfluidics. Opposed to the traditional closed-channel microfluidics, the new open-channel designs only feature three solid enclosing sides, while one side is either open entirely or covered with a fluid, e.g. oil. This brings several advantages, such as free physical access to the microchannel, with the oil phase reducing evaporation and contamination. A schematic of their open-channel device design is displayed in Fig. 8(B).

Another important aspect of the infection pathway is the germination of fungal spores. Suppressing this would stop the infection at the very first instance, thus clearing the pathogen before it has the chance to start any metabolic activity at the expense of its host, thereby minimizing harm to the patient. A full understanding of the mechanisms underlying spore germination would, therefore, significantly benefit antifungal medication. To precisely study these cellular processes, Barkal et al. (2016) designed a microfluidic device for high resolution germination assays with *C. neoformans*. Amongst others, they were able to demonstrate that common clinical fungicides do not inhibit spore germination, showing that in order to intervene at the germination stage, new methods need to be developed and established (reviewed in Bernier et al. (2022)).

### Lung-on-a-Chip

Some fungal pathogens target a specific organ rather than causing systemic infections. Especially for delicate organs like the lung, *ex vivo* models are useful to study the processes involved in the disease. Organ-on-a-Chip microfluidic technologies (Box 3), a subdiscipline within the field of microfluidics, have emerged in the last decades as a platform to replace animal and cell culture models in preclinical trials, thus making these trials more reliable and ethical (Beißner et al. 2016). Lung-on-a-Chip platforms have been used to investigate lung-related diseases and injuries over the last 15 years (reviewed in Shrestha et al. (2020) and Bennet et al. (2021)). With such microfluidic set-ups, it is possible to reconstruct the 3D architecture of the lung on a cellular level as well as simulate flow conditions and breathing motion (Huh et al. 2007, Huh 2015). Furthermore, pathogen–tissue interactions can be mimicked and precisely analyzed (Huh et al. 2010).

Box 3. **Organ-on-a-Chip**

The emergence of Organ-on-a-Chip technology, which stemmed from a fusion of the fields of tissue engineering and microfluidics, has been anticipated animatedly by the wider community of biological and medical scientists. This promising new approach aims to reconstruct parts of organs or tissues *in vitro* to obtain functional model systems, mimicking reality as closely as possible. Organ-on-a-Chip platforms combine 3D-cell cultures, grown with the aid of scaffolds, with fluid channel networks, e.g. simulating vascularization. Important for such a task is to understand the key physiological aspects, namely the parenchymal tissue function, tissue barrier properties, and interactions, e.g. endothelial-blood stream, epithelial-lumen, as well as the interplay with other organs (Zhang et al. 2018b). Organ models are of the utmost significance for drug discovery, both from an ethical and economical viewpoint, as they reduce the need for animal tests. From a medical standpoint, it is desirable to use human organ models for drug testing since results obtained with conventional 2D human cell cultures or animal models have a limited transferability onto the human anatomy and physiology. So far, this highly interdisciplinary methodology has been applied to model nearly all existing organs or tissues of the human body, such as the liver (Yoon No et al. 2015), kidney (Ashammakhi et al. 2018), and brain (Bang et al. 2019).

**Figure 9.**
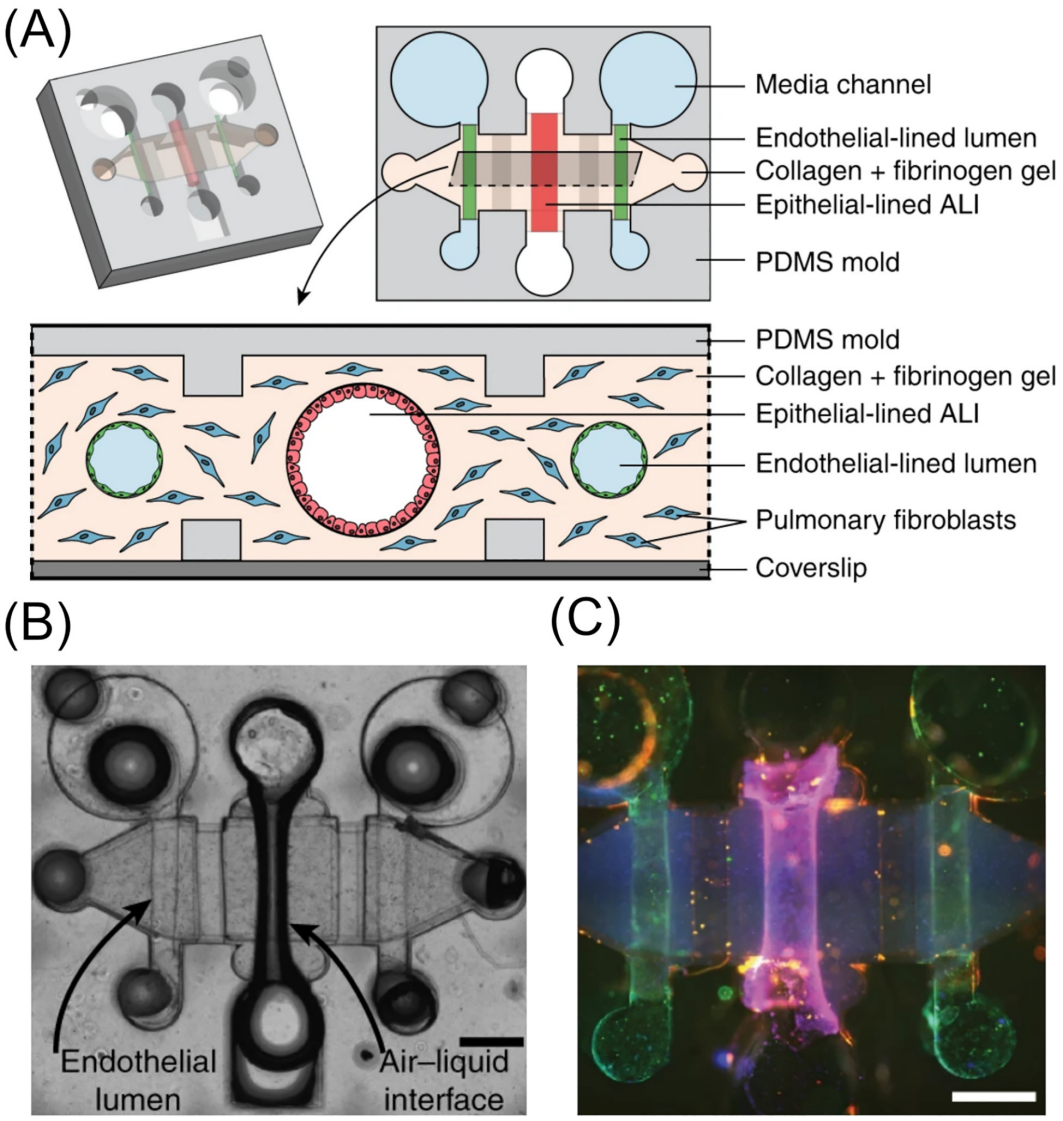
Lung-on-a-Chip device for studying fungal lung infections. (A) Schematic illustrating a microfluidic device used to mimic the human bronchiole. (B) Microscopy image of the experimental set up. (C) Channels coated with cells stained with Hoechst (blue, nuclear stain), anti-CD31 antibody (green, endothelial tight junction marker), and anti-EpCAM antibody (red, epithelial cell–cell adhesion maker). Scale bars are 500 µm. Images reproduced with modifications from Barkal et al. (2017) with permission from the Creative Commons Attribution license (www.creativecommons.org/licenses/by/4.0/).

Recently, this methodology has been applied to the study of fungal respiratory diseases, using the human fungal pathogen *A. fumigatus* as a model organism (Paolicelli et al. 2019). Barkal et al. (2017) developed a microfluidic model of the human bronchiole, including the vascular and airway compartments as well as the extracellular matrix (Fig. 9). They then studied the inflammatory response caused by *A. fumigatus* infection and later a tripartite interaction, including a volatile contact with *Pseudomonas aeruginosa*. In the tripartite system, the inflammation-related cytokine secretion was significantly elevated, compared to both the dual interactions, however, showed significant variations with regards to cytokine type. Further, it was possible to monitor leukocyte distribution in response to the infection. The detection of this complex multikingdom aspect of the infection was only made possible due to the development of a sophisticated and accessible platform, such as the presented device. Bacteria and fungi were physically separated, hence communication could only occur through volatiles diffusing through a collagen–fibrinogen gel matrix, just like in the real bronchiole. As a future improvement and extension of their platform, they suggest implementing an appliance to introduce airborne pathogens into the system, to better mimic the main real-life infection pathway, which would facilitate studies concerning the early stages of the disease.

## Investigations on filamentous fungi at the cellular level

In fungal infections with *C. albicans*, for instance, another stage in the infection cycle concerns the dissemination of the pathogen within the body. In the case of several *Candida* spp. this takes place in the filamentous form, with hyphae searching for susceptible cells and actively penetrating their membrane. To understand various aspects regarding the filamentous form of fungi, microfluidic techniques again provide a useful tool, where the possibility to design precise microstructures using established lithography techniques can be exploited to mimic naturally occurring habitats, not only for pathogenic fungi, but also soil or other filamentous fungi. This facilitates studies, which would otherwise simply not be possible. Creating artificial microenvironments to study fungal behaviour at different scales, from the hyphal to the cellular and even subcellular level should bring insights into biological processes and interactions that would otherwise take place within a ‘black-box’, i.e. within masked soil or organic tissue environments. As well as the physical elasticity of materials like PDMS, which can be exploited for force measurements, microfluidic device design can be specifically adapted to improve imaging, e.g. by guiding hyphal growth in a spiral, thus keeping the fungus in the focus area (Lee et al. 2016). A broad overview concerning microfabricated formats and their beneficial use for fungal research, with a focus on hyphal responses to microfabricated structures, can be found in a short review by Bedekovic and Brand (2021).

### Space searching and foraging strategies

Nicolau and colleagues have dedicated intensive research efforts to uncover hyphal behaviours of different filamentous fungi. Specifically, they created microchips containing different obstacles and maze structures to mimic certain aspects of the fungus’ natural environment at both the cellular (Held et al. 2009a,b,c, 2010a, 2011b) and subcellular level (Held et al. 2010b, 2019). These pioneering studies in the area have delivered new and important insights into both fungal life cycles and lifestyle.

When investigating the filamentous model fungus *N. crassa*, the authors found significant changes in its hyphal branching behaviour (e.g. frequency and angle). They observed an immediate onset and a lack of an adjustment period upon navigating through microfluidic network architectures (Held et al. 2010a). Interestingly, the hyphae were found to employ several strategies, depending on the type of obstacle they encountered. In general, *N. crassa* exhibited a directional memory, which was usually upheld after experiencing mechanically induced reformation events. Such events included ‘hit and split’ (i.e. the division of a mother hypha into two daughter hyphae) upon head-on collision with an obstacle, increased hyphal branching after passing through bottlenecks or active hyphal growth into openings through the ability to sense and identify these patterns (i.e. thigmotropism; Brand and Gow 2009, Held et al. 2011a). Occasionally, nestling was observed, meaning that the hyphae attached to the physical structure and were able to track it. This phenomenon was further studied by introducing micrometre-sized PS beads with epoxy-functionalized surfaces into the medium (Held et al. 2011b). By subjecting hyphae to different densities of the coated beads and comparing the results to the behaviour of the hyphae observed in their bead-free microfluidic systems, they found the attraction to be dependent upon the chemical property of the surface rather than being purely mechanically induced. They hypothesized an electrical attraction between the negatively charged bead surface and the positively polarized apex of the hypha, the latter due to a high concentration of calcium ions. With the generally less negatively charged and hydrophilic PDMS walls, this was not observed. This set up has great potential for further exploitation with other chemical moieties attached to the beads, or a direct functionalization of the microdevice channels, opening new avenues for the creation of more complex microenvironments. The involvement of cellular microtubules and the Spitzenkörper, the apical body present in many filamentous fungi, in hyphal space navigation and thigmotropism were later studied in more detail. According to their findings, the Spitzenkörper acts in a manner analogous to that of a gyroscope, which is stabilized by the microtubule scaffold; indeed, both structures are understood to be major players in directional memory. The microtubules are strictly aligned along the growth axis in the apical region, while less strict and rather randomly directed and distributed in the subapical regions. Furthermore, they discovered two distinct mechanisms related to hyphal space searching, specifically directional memory and obstacle-induced branching, with the former found to be the most effective and the latter implemented as a back-up strategy in *N. crassa* (Held et al. 2010b, 2019). Additionally, the acquired data on fungal space searching strategies was used to develop bioinspired computational algorithms to solve certain problems more efficiently. This could be beneficial for the design and programming of nanosensors or nanomachines (Hanson et al. 2006, Nicolau et al. 2006, Asenova et al. 2016).

**Figure 10.**
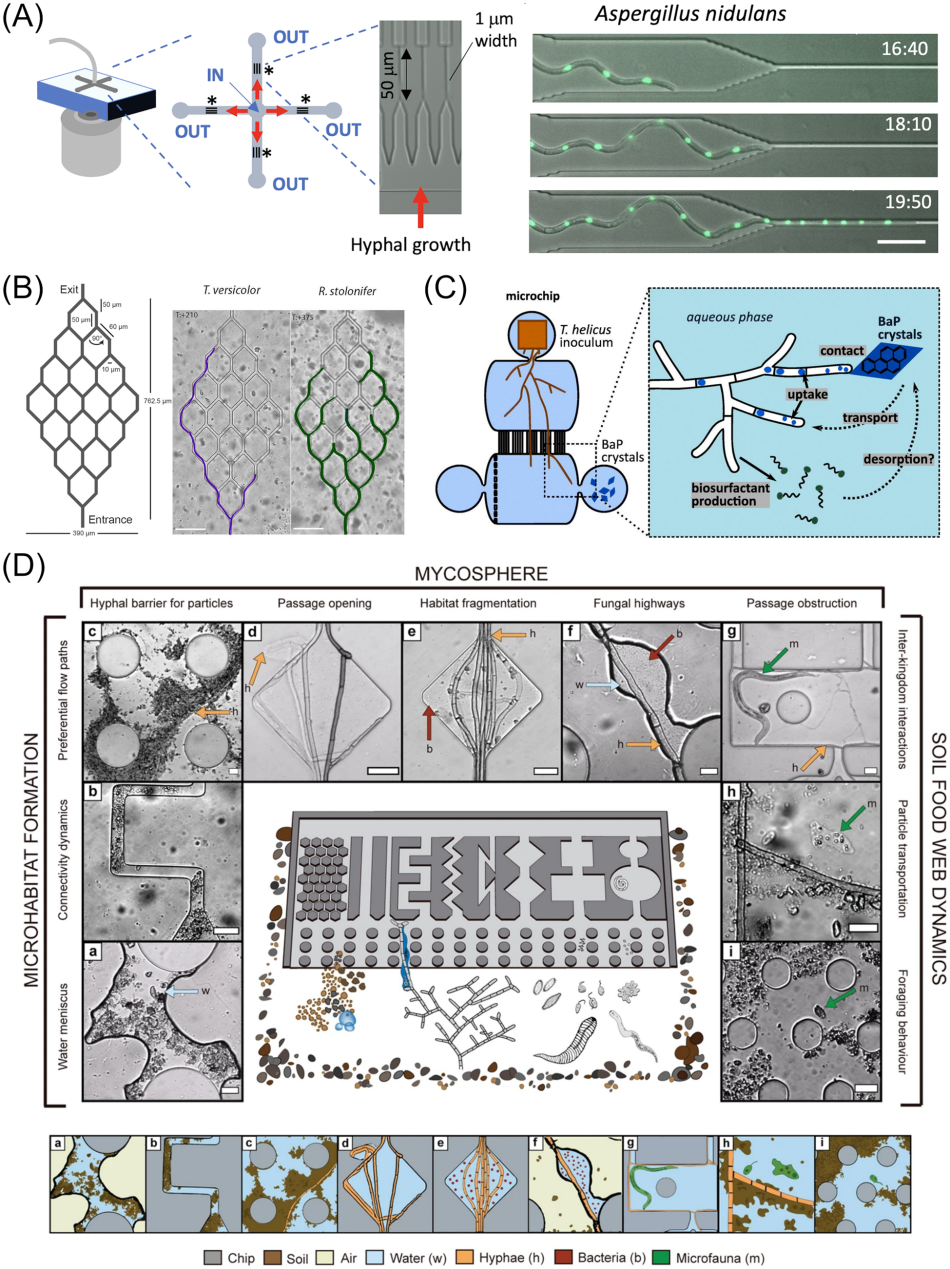
Microfluidic platforms for the study of fungal behaviour in artificial microenvironments mimicking aspects of the natural habitat. (A) Device used to study the behaviour of filamentous fungi upon facing special confinement on a subcellular level. Scale bar represents 20 µm. (B) Microdevice for studying space searching strategies in different fungal species. Scale bars represent 100 µm. (C) Microchip used to investigate chemotropism and uptake of lipophilic molecules (e.g. benzo[a]pyrene, BaP) by filamentous fungi. Image reproduced with modifications from Baranger et al. (2021) with permission from Elsevier (Licence Number: 5271511231788). (D) Microfluidic platform simulating several aspects of soil communities, featuring different channel designs filled with soil particles, soil bacteria, microfauna, and filamentous fungi, as well as being saturated with water or with air. Scale bars represent 20 µm. Images in (A), (B), and (D) reproduced with modifications from Fukuda et al. (2021), Hopke et al. (2021), and Mafla-Endara et al. (2021), respectively, with permission from the Creative Commons Attribution licence (www.creativecommons.org/licenses/by/4.0/).

Inspired by these pioneering studies, several other researchers followed this path and designed new microfluidic chips for comparative studies with other fungi, such as *Armillaria mellea* (Held et al. 2009b), *Talaromyces helicus* (Baranger et al. 2020), and *Cladosporium macrocarpum* (Podwin et al. 2021), which revealed strong differences between species. These observed variations were then systematically correlated with their habitat, preferred foraging style, and overall growth behaviour. Fukuda et al. (2021) especially focussed on the effect of bottleneck-like structures (i.e. spatial confinements smaller than that of the hyphal diameter) upon hyphal growth (Fig. 10A). They found mainly two outcomes, based on which they grouped different fungal species. While fast growing fungi, such as *Rhizopus oryzae* or *N. crassa*, are forced to stop by the confinement or depolarize, meaning they bulge at the apex of the hypha and loose directional growth altogether, the hyphae of slower growing fungi, e.g. *A. nidulans* or *Fusarium oxysporum*, remain polarized and continue growing undisturbed after exiting the confinement. To explain this phenomenon, a correlation was drawn between the number of vesicles present in the hyphal cytoplasm, turgor pressure, and the ability to adapt to confined growth. A high number of vesicles and high intracellular pressure aids a fast growth with extensive branching; however, when entering a confinement, these attributes lead to an excess of vesicles and pressure, which eventually disrupts the directional memory (usually sustained by the Spitzenkörper) or results in complete growth arrest. On the other hand, slow growing fungi are well-equipped to manoeuvre through confined spaces. Which of these modes the fungi use, correlates with their overall growth and survival strategy.

A similar, but broader, categorization of filamentous fungi and their space searching capability was conducted by Aleklett et al. (2021), who took additional aspects into account. With their Obstacle Chip, which features a vast array of different parallel obstacle channels, they studied seven filamentous soil fungi based on their branching behaviour, growth velocity, foraging style, hyphal shape, and mycelial density. It was found that the fungi analyzed in their study can be categorized into two main groups: (i) the guerrilla strategists, developing few and short-lived, but long-reaching, fast growing hyphae and (ii) the phalanx strategists, forming dense, strong hyphal mats. This interspecies variation in growth pattern is, from an evolutionary and ecological standpoint, important for the exploration of all available niches within a biosphere/ecosystem. Similar findings were obtained by Hopke et al. (2021) in their crowdsourced study, aptly named the ‘Fungus Olympics’ (Fig. 10B). Here, some fungi were found to navigate faster through certain structures, while others trumped them in other ‘disciplines’, depending on their branching strategy. These studies have demonstrated how microfluidic technologies can help to unravel and investigate the behavioural patterns that these fungi exhibit in their natural environment, which are otherwise barely detectable. The most recent study in this field, conducted by Mafla-Endara et al. (2021), attempted to move even closer to the natural habitat by introducing abiotic and biotic factors into their microfluidic device. Besides physical obstacles, possessing different sizes and shapes, the Soil Chip contains real soil particles, water, and air, as well as fungi, bacteria, and protists. Thus, they projected the ecosystem soil environment, with numerous different niches, onto a small, imageable microfluidic platform (Fig. 10D). Amongst others, they were able to observe common events like soil particle relocation or accumulation, passage opening or obstruction, and thus habitat fragmentation, which are all processes that fungal hyphae are frequently involved in. Furthermore, they observed interactions between hyphae and other organisms, mainly bacteria. In line with previous discoveries and the novel concept of ‘fungal highways’ (Kohlmeier et al. 2005, Warmink et al. 2011), it was observed that bacteria strongly benefit from, or even rely upon, fungal hyphae to access and acquire nutrients, either by navigating through demanding soil structures, e.g. crossing air gaps, or assisting in the decomposition of organic or inorganic substances. The latter, however, also works *vice versa*, with fungi relying on bacterial enzyme activity for nutrient uptake (Jiang et al. 2021). Another important trait of filamentous fungi relates to their ability to transport and disperse water, together with soluble compounds, along their hyphae, an important aspect not only for bacteria, but the soil community as a whole (Mafla-Endara et al. 2021). The hyphal interactions discussed thus far have further been subject to more focussed studies of specific aspects of this complex ecological interplay. Specifically, compartmentalized microdevices can be used to investigate how fungi explore niches containing isolated substances. Baranger et al. (2021) studied how the soil fungus *T. helicus* forages for spatially isolated benzo[α]pyrene, an example of a polycyclic aromatic pollutant (Fig. 10C). Interestingly, they discovered that the fungus employed active mechanisms, involving the excretion of biosurfactants, to reach, decompose, and uptake this hydrophobic substance. These abilities could be exploited further as part of a bioremediation strategy, where the fungus would help to degrade lipophilic environmental toxins.

### Hyphal tip force measurements

Hyphal tips can penetrate a range of host tissues, ranging from plant cells for symbiosis establishment (Parniske 2000), to insect cuticles (Gabriel 1968) and human cells in infectious disease (Fernandes et al. 2018). Even more impressive, hyphal tips can penetrate mineral substrata by digging into mineral grains or at the interface between grains (Landeweert et al. 2001). Microfluidic experimental systems enabling hyphal tip force measurements at the single-cell level are, therefore, evolving, with PDMS being the material of choice for these types of studies. Not only is PDMS permeable to gases, widely inert, biocompatible, transparent, and easily patterned using simple photolithography methods (Box 1), other exploitable aspects concern its elasticity (see also the ‘Pressure-based active trapping in single-cell studies on yeasts’ section) and ability to adjust the tensile strength of the polymer, simply by changing the ratio of PDMS base and curing agent. This, therefore, presents the opportunity to conduct tip force measurements without the need for expensive sensors. Just by imaging the deformation of a PDMS structure by a hyphal tip in a microfluidic device, the force can be calculated using the known Young’s modulus for a mechanical deflection model (Ghanbari et al. 2012).

To further investigate infection mechanisms and pathogenicity in *C. albicans*, Thomson et al. (2015) utilized PDMS soft lithography to manufacture microdevices with several obstacles. The fungal hyphae had to navigate around these structures and were found to exhibit different behaviours. Furthermore, because of the known and adjustable tensile strength of PDMS, the force exerted by the hyphal tips could be calculated and analyzed. The measured forces of around 9 µN fall within the range of tensile strengths found in common cell walls and membranes. In agreement with studies from other groups, they hypothesized that the fungus executes two different modes of cell wall and membrane penetration. Depending on the toughness of the tissue, *C. albicans* is able to penetrate the barrier with pure mechanical force, while for others, an additional loosening of the wall with enzymes is necessary, involving complex cellular and morphological reorganization (Puerner et al. 2020). Alternatively, the fungus is able to navigate around impenetrable barriers (Thomson et al. 2015).

**Figure 11.**
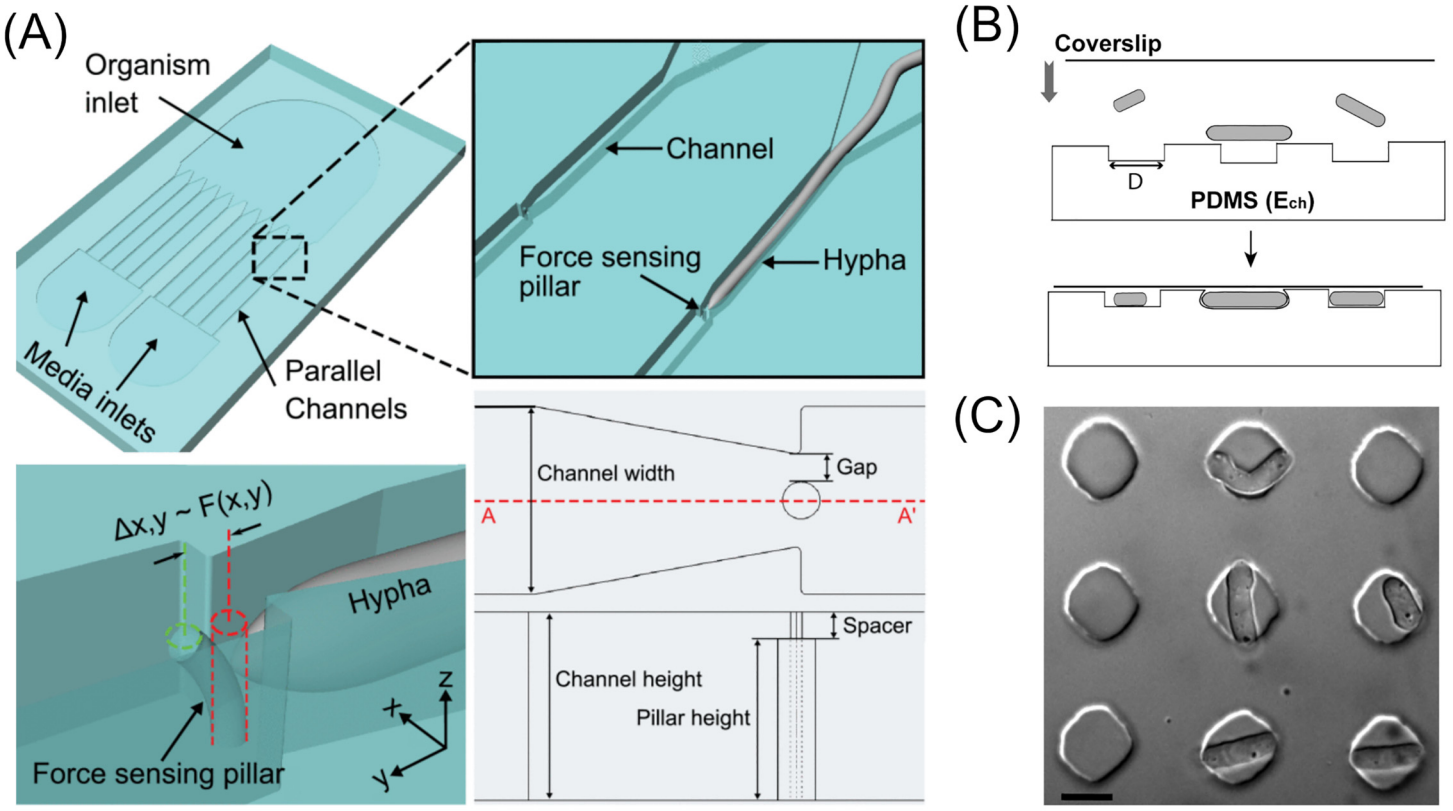
Microfluidic devices for measuring hyphal and cellular tip force. (A) A channel design containing a dead-end channel with a force sensing pillar. Upon deflection of the pillar, values for the force exerted by the hyphal tip can be calculated. Image reproduced with modifications from Sun et al. (2018) with permission from the Creative Commons Attribution license (www.creativecommons.org/licenses/by/4.0/). (B) Microwells precisely fitting single cells of *S. pombe*. Upon cell fission and growth, the elastic PDMS wells are deformed and forces exerted can be deduced. (C) Microscopic image of trapped yeast cells with clearly visible deformation of the wells upon cell growth. Scale bar represents 10 µm. Images (B) and (C) reproduced with modifications from Minc et al. (2009) with permission from Elsevier (Licence Number: 5271520737780).

Similarly, Sun et al. (2018) designed a microdevice with force sensing pillars to measure protrusive forces exerted by *N. crassa* as well as the oomycete *Achlya bisexualis* (Fig. 11A). Oomycetes are fungus-like eukaryotes and are comparable to fungi in many ways, in particular for some species, their filamentous form and exploratory nature. Furthermore, many strains of the phylum Oomycota are pathogenic to fungi and plants (Beakes et al. 2012). Therefore, mycology and oomycete research are closely related. With their micropillar arrays, they measured protrusive forces of maximal 11 µN and 7 µN for *N. crassa* and *A. bisexualis*, respectively. When deflected by the pillar, the oomycete hypha was able to exert a bending force of up to 19 µN (Sun et al. 2018). Using another platform, they optimized the trapping of single zoospores of *A. bisexualis* with a sophisticated valve system to study forces exerted by germ tubes upon germination. The measured forces ranged between 0.73 and 1.66 µN, which is slightly less compared to the forces of adult hyphae (Sun et al. 2020).

Minc et al. (2009) exploited the elastic characteristics of PDMS for a slightly different application, i.e. to measure forces developed by growing cells of the fission yeast *S. pombe*. They trapped the rod-shaped cells in microwells having a diameter of between 10 and 50 µm and calculated the forces from the deformation of the wells upon stretching and buckling of the cells (Fig. 11B and C). Interestingly, the calculated forces exerted by the yeast cells were around 11 µN, which is within the range of the previously discussed forces by *N. crassa* and *A. bisexualis*. Further, they studied the underlying intracellular mechanisms of the force generation and found that mainly turgor pressure was involved, which was actively regulated with enzymes such as glycerol-3-phosphate dehydrogenase. This enzyme catalyses the synthesis of glycerol, the main component determining turgor pressure. However, they assumed that the growth force is independent of actin cable organization, which is generally important for polarized cell growth, after comparing their findings with the behaviour of a mutant strain lacking actin cables, *for3Δ*.

### Hyphal interactions

Besides the mechanical aspects influencing filamentous fungal growth and behaviour, another avenue that the microfluidic approach allows is the investigation of biotic factors, i.e. inter- or intraspecies interactions. By designing special microfluidic devices with channels, chambers, and perfusion facilities, this provides a means to precisely trigger and analyze interactions between bacteria and fungi (Stanley et al. 2014, Uehling et al. 2019), as well as fungal–nematode (Schmieder et al. 2019), fungal–virus (Ghanem et al. 2019), and fungal–fungal (Stockli et al. 2019) interactions.

#### Bacterial–fungal interactions

**Figure 12.**
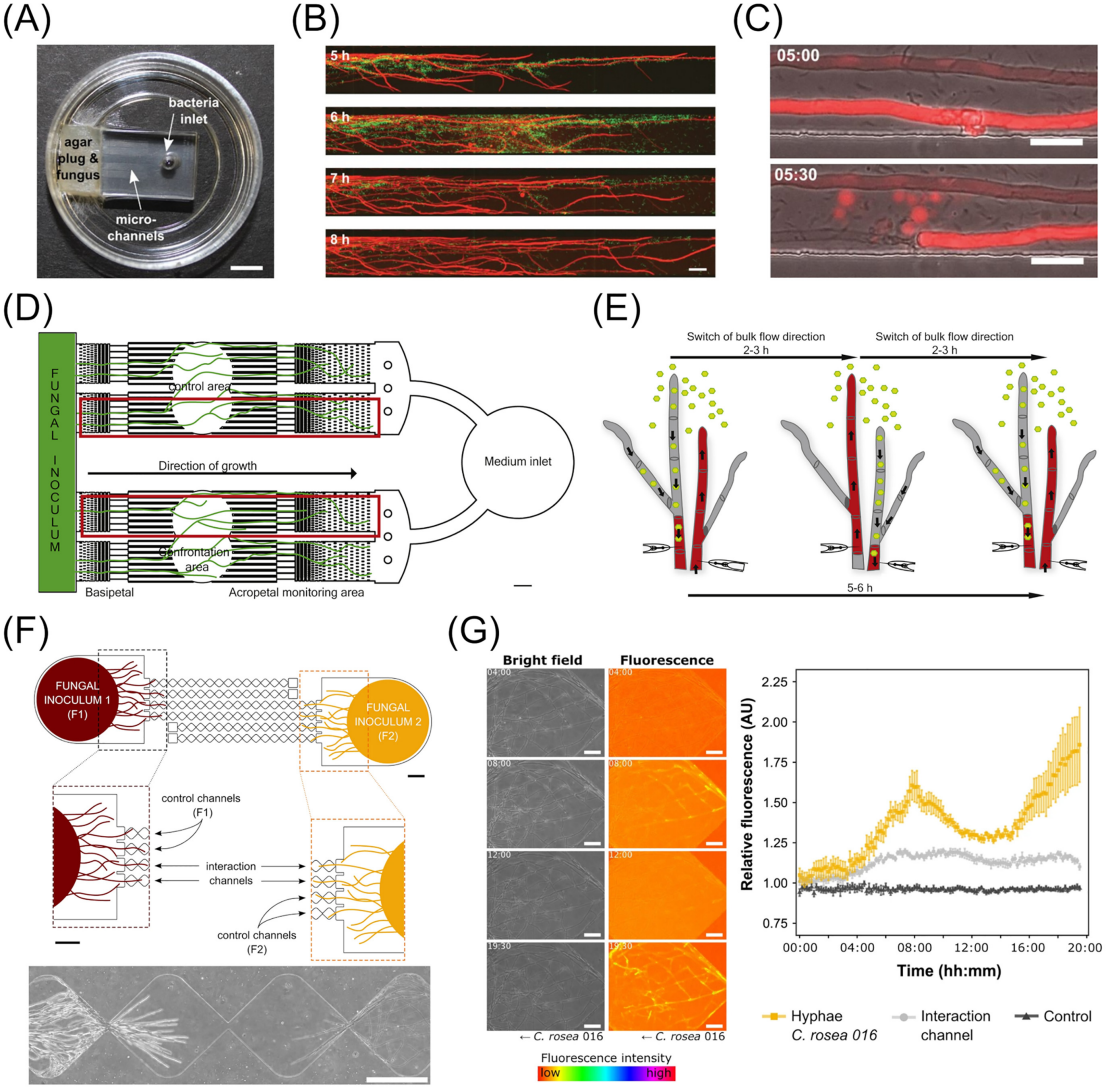
Microfluidic devices for investigating hyphal interactions. (A) Image of the bacterial–fungal interaction (BFI) device, inoculated with an agar plug containing fungal mycelium. Scale bar represents 5 mm. (B) *Bacillus subtilis* (green) attaching to *Coprinopsis cinerea* hyphae (red), coinoculated in the BFI device. Scale bar representing 50 µm. (C) Time series showing the collapse of a *C. cinerea* cell and subsequent loss of its cellular contents, caused by *B. subtilis*. Scale bar representing 25 µm. Images (A)–(C) reproduced with modifications from Stanley et al. (2014) with permission from Oxford University Press (Licence Number: 5297210130322). (D) Schematic design of a device for studying fungal–nematode interactions. Scale bar represents 500 µm. (E) Proposed model for fungal response upon nematode predation. Bulk hyphae switch cytoplasmic bulk flow direction every 2–3 h in order to redistribute nutrients (e.g. 2-Deoxy-2-[(7-nitro-2,1,3-benzoxadiazol-4-yl)amino]-D-glucose (2-NBDG), green hexagons). Red indicates the spatial distribution of the *C. cinerea* defence response upon predation. Images (D) and (E) reproduced with modifications from Schmieder et al. (2019) with permission from Elsevier (Licence Number: 5297260732132). (F) Schematic design of the fungal–fungal interaction (FFI) device (top). Scale bar represents 1 mm. (Bottom) Phase contrast image of hyphae of *C. rosea* and *F. graminearum* approaching each other in the interaction zone. Scale bar represents 250 µm. (G) Time series showing the detection of GFP within the mycelium of *C. rosea* (left). The fluorescence signal within these hyphae was found to increase and decrease compared to control channel (right). Images (F) and (G) reproduced with modifications from Gimeno et al. (2021) with permission from the Creative Commons Attribution license (www.creativecommons.org/licenses/by/4.0/).

The first investigations involving the interaction of fungi with other microbial species at the single-cell level were dedicated to the study of bacterial–fungal interactions (BFIs). Stanley et al. (2014) developed a microfluidic device, termed the BFI device (Fig. 12A), specifically designed to accommodate fungal hyphae and facilitate coinoculation with bacteria through a separate inlet. The interaction between the fungus *Coprinopsis cinerea* and the bacterium *Bacillus subtilis* was investigated in this study, where it was observed that inoculation with the wild-strain (*B. subtilis* NCIB 3610) was followed by hyphal growth arrest, while the domesticated *B. subtilis* 168 laboratory strain did not exhibit this activity. Using the BFI microfluidic device, it was then possible to gain a deeper understanding of BFIs in real-time and at the cellular level. In turn, it was revealed that bacteria attached to specific subsets of hyphae, regardless of whether they were wild- or laboratory strain, or even dead bacterial cells (Fig. 12B). Additionally, this suggested the existence of hyphal differentiation within the mycelium. The hyphal growth arrest was found to coincide with the collapse of some hyphal compartments and the subsequent formation of blebs, exocytotic vesicles still containing the fluorescent marker for up to several hours after the collapse (Fig. 12C). In a further experiment using a second microfluidic device, termed the fluid exchange device, they perfused the hyphae with bacterial supernatant and lipophilic extracts. Since the effect on the hyphae was analogous to that observed with the bacteria, it was postulated that the fungicidal activity derived from lipophilic exudates rather than active bacterial enzymes.

To protect themselves against such bacterial attacks, fungi have developed antibacterial strategies. With microfluidic technology it is possible to single out hyphae and subject them to bacterial supernatant and extracts with subsequent precise imaging of the resultant effect, yielding important information for basic research as well as for practical applications, like pest control methods (Stockli et al. 2019). Harting et al. (2021) investigated the antagonistic relationship between certain *Verticillia* spp., pathogenic fungal species of important crops, and *Pseudomonas* spp. With the help of a microfluidic platform, they analyzed the fungal–bacterial interaction on a cellular level, granting insight into the cellular and molecular processes involved and how the bacteria inhibit fungal growth and plant colonization.

Similarly, mutually beneficial interactions can be investigated in microfluidic devices. The above featured BFI device was further employed to study the endosymbiosis between the fungal plant pathogen *Rhizopus microsporus* and the bacterium *Burkholderia rhizoxinica*, detailed in a preprint by Richter et al. (2020). Uehling et al. (2019) investigated the (pre-) symbiotic signalling between the fungus *Mortierella elongata* and the bacterial *Burkholderia* strain BT03. Their studies revealed a multistage bidirectional communication involving exudates. The first stage is marked by exudation of organic acids, such as erythronic acid, by the fungus, which are then recognized and used by the bacteria. The second stage involves a response signal (yet to be determined) sent out by the bacteria, which enhances hyphal growth of the fungus. The specificity of the signals is assumed to increase from stage to stage, though subsequent stages are so far not fully discovered. The group further set out to develop an easy-to-use microfluidic device in an attempt to democratize the microfluidic tools available for the bacterial–fungal research field. Their devices come premade, sterile, and vacuum packaged. Besides the obvious benefit for protection and conservation of the device during transport, with the help of the vacuum, channels fill automatically with the desired medium or solution within minutes after opening the sealing, thus avoiding problems with priming. To test the device’s performance in the field, the group sent out samples to labs all over the world. In general, reports came back demonstrating successful application of the system to studies on fungal biofilms, fungal highways, BFIs, as well as a few investigations involving plant seeds or even neurons (Millet et al. 2019). This highlights the importance of designing microfluidic platforms that are amenable for use by nonexperts, thus encouraging adoption and use of the technique by mycologists.

Another study demonstrating the flexibility of microfluidic devices was conducted by Ghanem et al. (2019). They investigated and quantified the retention and distribution of bacteriophages along fungal hyphae. This aspect of soil ecology has so far received little attention, although partly due to the fact that investigations in this area are far from trivial. The retention of phages by hyphae was found to be up to 93%, in the case of the hydrophobic T4 phage and hydrophobic *C. cinerea* hyphae. Complemented with calculated surface interaction energy values, their findings have significant implications for environmental as well as medical questions.

#### Fungal–fungivorous interactions

Besides the world of microbes, soil fungi also regularly encounter slightly bigger, fungivorous organisms, such as protists and microfaunal organisms, which they must defend themselves from. In a two-part study, using a device based on the BFI device, Schmieder et al. (2019) investigated the distribution of locally administered nutrients throughout the hyphal network of *C. cinerea* as well as response mechanisms upon attack by the nematode *Aphelenchus avenae* (Fig. 12D). The group followed up on and confirmed previous findings on specialized hyphae and dynamic responses within the mycelial network. With the aid of a fluorescent glucose analog, 2-NBDG, and high-resolution fluorescence microscopy they monitored bidirectional transport of cytoplasmic nutrients and found periodical switching of the direction every 2–3 h in certain specialized hyphae (trunk hyphae) (Fig. 12E). Further, it was hypothesized that the flow dynamics are mainly controlled by septa, which can open and close their pores reversibly. While nutrient distribution is rather global, intending to supply all parts of the mycelium equally, even if nutrient sources are locally restricted, defence mechanisms tend to be only activated at the immediate site of attack. The defence response against the nematode is very specific to the fungivore, with two effector promotors, cgl2p and cctx2p, mainly involved. The defence programme was only activated in areas in direct contact with *A. avenae*, but was, however, quickly propagated in the trunk hyphae. In a continuation of the study, the group focussed further on the genes involved in the defence response, using a slightly modified microfluidic platform. From a channel opening in the confrontation zone, it was possible to extract fungal mycelium for downstream analyses (i.e. PCR and poly(A)+ RNA sequencing), thus directly analyzing the transcriptome linked to the interaction with the nematode. They were able to identify 1229 differentially expressed genes; until this point, only 37 genes had been discovered using conventional plate-based techniques. Their results further show an involvement of antibacterial defence genes, suggesting the interaction to be a tripartite (fungus, nematode, and nematodal microbiome) instead of a bipartite one, which is true for many interactions in nature (Tayyrov et al. 2019).

#### Fungal–fungal interactions

Very recently, Gimeno et al. (2021) developed a microfluidic platform to investigate antagonistic fungal–fungal interactions (FFI) between the plant pathogen *F. graminearum* and biocontrol agent *C. rosea* (Fig. 12F). In the course of these investigations, further and more detailed insights into their antagonistic interaction was gathered. In particular, a loss of fluorescence in GFP-tagged *F. graminearum* was observed to coincide with the detection of GFP in the mycelial network of the predator, allowing to postulate an uptake and redistribution of material from the attacked fungus to the attacking fungus (Fig. 12G). In the future, the presented FFI device is anticipated to contribute further to revealing deeper insights into the mechanistic details of FFIs at the single-hyphal level, information needed for the development of novel and much needed strategies to control fungal pathogens in agriculture.

## Future perspectives and concluding remarks

Lastly, we provide a future perspective and concluding remarks detailing a number of new frontiers in which microfluidic technology is expected to aid future research in this field of study, with a focus on AMF, nuclei behaviour in filamentous fungi, multispecies interactions, and electrical measurements for cell-to-cell communication.

### AMF

When considering the study of soil microbes and interspecies interactions with fungi using microfluidic methods, mycorrhizal fungi, such as the obligate symbiotic AMF, call for inclusion. Forming one of the most important fungal–plant symbioses in nature, AMF interact with plant roots, providing mineral nutrients and water in exchange for carbon and lipids. Specifically, a key element of this mutualistic liaison is the plant’s contribution of photosynthesis products in exchange for soil nutrients, mainly P (up to 90%) and N, but also Zn, Cu, and Fe (Smith and Read 2010). Furthermore, the mycorrhizal association contributes to an improved resilience against biotic and abiotic stresses in the plant, such as increased resistance against below and above-ground pathogens, and tolerance to trace elements and hydrocarbon pollutants or drought and salinity, as well as a reduction of nutrient leaching and an improved environment for the establishment of fresh seedlings (van der Heijden 2004). During the last decades, therefore, AMF have been subject to intensive research, with the main drive towards agricultural exploitation of the advantages. Here again, the soil-borne and obligate nature of these fungi and their symbiosis renders most studies ‘black-box’ experiments, with microfluidics representing a promising tool to lift this veil. Indeed, so far only a single publication has surfaced in this area, which focusses on AMF spore sorting (Srisom et al. 2020).

Besides giving insight into their hyphal thigmotropism and space searching strategies, the development of novel microfluidic technologies could help solve prominent questions that have been baffling researchers for decades. Much is still unknown about AMF hyphae morphogenesis and polarized growth of the extraradical mycelium. AMF develop an intricate network of hyphae that consists of runner hyphae, that ramify into secondary and tertiary hyphae, for example, and ultimately form the branched absorbing structures involved in the uptake of nutrients (Bago et al. 1998). A mathematical model used to simulate nutrient uptake was described in Schnepf et al. (2011), which provides a foundation for studies involving single hyphae at the cellular level within microfluidic systems. AMF hyphae also interconnect individual plants of the same or different species to form ‘common mycorrhizal networks’ (CMNs; Simard et al. 2012). CMNs can transfer nutrients between plants or warning signals in response to attacks by fungal pathogens and insect pests, thereby acting as below-ground interplant defence communication tools (Babikova et al. 2013). Crucial questions about the specificity of the signals regarding the species of plants or pests, and the nature of the signal(s) transferred, need to be resolved. In addition, other molecules, such as small RNAs, and virus genomes might be transported through CMNs, with important implications for plant health and crop protection (Wang et al. 2017). The development of new microfluidic tools can help to understand the multitude of functions of hyphae and CMNs, taking the science well beyond the current state-of-the-art and, thus addressing global challenges such as food security and ecosystems management. One major challenge yet to address is the still-to-be-firmly demonstrated presence of the Spitzenkörper, a structure found at the hyphal tip that mediates hyphal growth direction and hyphal tip morphology by the delivery of secretory vesicles. These apical vesicular clusters have been suggested in AMF, although not firmly demonstrated (de la Providencia et al. 2005). These structures are associated with actively growing hyphal tips and, thus in the 3D architecture of extraradical mycelium, presumably being involved in the process of anastomosis formation and the hyphal healing mechanism (HHM), two important mechanisms of hyphal fusions between compatible mycelia evolved by AMF to increase their chances of survival. Curiously, anastomoses and HHM differ markedly between species belonging to the Gigasporaceae and Glomeraceae, two of the globally most predominant AMF families, suggesting divergent strategies for the colonies to explore and exploit their environments and create large networks (Voets et al. 2006). Recently, a negative impact of azoxystrobin and fenpropimorph, two active ingredients widely used in agriculture to protect crops from fungal diseases, was noticed on the HHM. In the presence of these compounds, contact and hyphal fusion are disturbed (Rodriguez-Morelos et al. 2021). One hypothesis is the possible perturbation of the Spitzenkörper, or sterol content and lipid rafts. Microfluidic platforms could thus help in the real-time observation of anastomoses and HHM (e.g. time series describing hyphal–hyphal fusion), but also in understanding the mechanisms involved in the polarized growth of hyphae and development of ‘Wood Wide Webs’ (Rhodes 2017) and CMNs, how changes in the environment disturb this essential mechanism involved in mycelium integrity and functioning, as well as AMF ecology, genetics, and evolution.

Another major avenue that can be approached with microfluidic technology development is the exploration of the wide variety of social interactions, including cooperation (e.g. mutualism, altruism, selfishness, and spite), that microorganisms have developed to survive and prosper in nature. In particular, the role of AMF hyphae in recruiting microbes into their hyphosphere, the so-called second genome of AMF, has become the center of attention of numerous studies (Zhang et al. 2022). Until the recent decade, the AMF hyphosphere has been overlooked mainly due to the difficulty of studying AMF–bacteria interactions on the surface of hyphae in a context free of any microorganisms other than those targeted (Duan et al. 2022). Fortunately, *in vitro* cultivation of AMF on root organs or whole plants has enabled the production of root-free compartments containing large amounts of hyphae devoid of any undesirable contaminants to study the interaction between AMF and phosphate solubilizing bacteria (PSB) or communities of bacteria growing on the surface of hyphae (Jiang et al. 2021, Zhang et al. 2018b). Using these systems, it has been shown that hyphae of AMF can selectively recruit PSB in the hyphosphere and reward them with hyphal exudates (Zhang et al. 2018b), which is a tangible sign of mutually beneficial cooperation between both partners, the so-called ‘direct reciprocity’ [‘*individuals will tend to help those who help them*’*—*Roberts (2008)]. Microfluidic technology could help to explore the intimate relationship/interactions occurring at the hyphal surface using, amongst others, brightfield and fluorescence microscopy techniques. Interaction studies on the hyphal surface can also concern pathogenic bacteria or even tripartite scenarios involving hyphae/beneficial bacteria/pathogenic bacteria. For instance, the yet unexplored indirect reciprocity [‘*I help you and somebody else helps me or does not harm me*’—Nowak and Sigmund (2005)] in the cooperation between AMF and bacteria can be explored using microfluidics.

A third important challenge related to the study of AMF regards understanding the reason as to why a restricted number of plant species (an approximation of 29%) including the plant model *Arabidopsis thaliana* are considered nonhost for AMF. Intriguingly, under some conditions, nonhost plants can become colonized by AMF and develop rudimentary AM phenotypes (Cosme et al. 2018). Microfluidic technologies could assist in elucidating the underlying reasons for these incompatibilities, e.g. by exploiting the capabilities of RootChips (Grossmann et al. 2011, Stanley et al. 2018).

### Nuclei behaviour in filamentous fungi

One particular trait of filamentous fungi regards the presence of multiple nuclei within a single cell compartment (Maheshwari 2005). This is particularly true for coenocytic fungi such as Mucoromycota, but Dikarya fungi also experience this state, either after sexual cell fusion or in a parasexual cycle. In addition to this, many fungi form anastomoses between individual hyphae to reorganize their network (Chagnon 2014, Croll et al. 2009, Read et al. 2009), which eventually leads to a redistribution of nuclei between different parts of a fungal colony, but also between distinct ‘individuals’ (though what exactly a fungal individual may relate to is debatable, given that fungi are considered modular organisms; Andrews 1995). These peculiarities come with the coexistence of genetically different nuclei in a single cell compartment, a state called heterokaryosis (Scott et al. 2019, Strom and Bushley 2016). The rules that govern nuclei exchanges, how genetically divergent nuclei coexist and compete in a cell space, and how this impacts the biology of filamentous fungi are all open questions within the field. Following nuclei circulation and exchange within the mycelial network of one fungus or witnessing nuclei exchange between different networks could be investigated using microfluidic technologies. This in turn would open new avenues for the understanding of fundamental aspects of fungal biology such as self/nonself recognition mechanisms (Kahmann and Bölker 1996) or sexual compatibility (Nieuwenhuis et al. 2013). Such knowledge would also be highly relevant for selecting fungi for biotechnological purposes not at the strain level, but rather at the nucleus level.

### Multispecies interactions involving fungi

Tailored microfluidic devices have started to reveal the complexity of interactions established by filamentous fungi with other soil dwellers, as detailed previously. As a result, given the diversity of organisms in the environment, still a myriad of additional relevant interactions could be investigated in detail using microfluidic platforms. Many of these interactions result from the exploratory behaviour of filamentous fungi (Aleklett and Boddy 2021) and their ability to construct an ever-changing network of hyphae that connects soil habitats throughout water unsaturated soil patches. This has led, for instance, to the observation of bacteria exploiting this network for their dispersal using the so-called fungal highways (Kohlmeier et al. 2005). Such dispersal paths in soils have been shown to be important for soil biogeochemical processes (Martin et al. 2012, Sun et al. 2019), but also for the promotion of genetic exchange (Berthold et al. 2016) and the encounter of bacterial viruses (bacteriophages) with their host populations (You et al. 2022). Interestingly, fungal highways also promote water redistribution in unsaturated systems, leading for instance to the germination of dormant bacterial cells (Worrich et al. 2017). However, so far, none of these processes has been witnessed with the cell-to-cell resolution that microfluidic technologies can offer. Although the use of fluid-filled devices could be counterintuitive for investigating interactions that depend on the level of water saturation within an environment, fungal colonization of dry and nutrient deficient microfluidic devices leads to water redistribution in the channels, which can be combined with cocultures of interacting organisms (Gimeno et al. 2021). In this way, the rules governing the transport of bacteria along fungal highways (directionality, growth, and motility trade-offs or impact of fungal cell wall remodelling), the mechanics of displacement (speed, effect of chemoattractants, or chemorepellents) or even the characterization of the liquid layer on the surface of fungal hyphae (composition, thickness, and viscosity) can be investigated. A better understanding of these processes can contribute to improving the design of biotechnological approaches in which fungal highways can be relevant, such as in soil bioremediation (Wick et al. 2007) or the biocontrol of soil-borne pathogens.

Fungi also participate in beneficial associations with algae or cyanobacteria, or even both together, to form lichens (Grimm et al. 2021). As microfluidic technology development allows researchers to design tailored microarchitectures to accommodate and guide interspecies interactions precisely, artificial reconstruction of lichens could contribute immensely to further understanding this composite organism. Á la Organ-on-a-Chip technologies, reconstructing lichen architecture within multilayered systems with controlled perfusion could allow investigations regarding symbiosis establishment and underlying mechanisms associated with the partnership. Moreover, the ability to procure these pioneering organisms on demand could be used to repopulate destroyed ecosystems.

### Electrical measurements in fungi

Furthermore, fungal mycelia are known to span vast networks in soil, connecting plants, and thus potentially acting as a ‘central connecting system’ within soil ecosystems. The communication mechanisms within the system are still poorly understood, but the possibility of having electrical currents as a means of fast information transfer would provide a system akin to a central neuronal system that could result in a coordinated behaviour required in such a complex system as a soil. Electrical currents have been shown not only to be important for the coordination of cell behaviour at all biological levels, i.e. from bacteria to humans (Brenner et al. 2006, Canales et al. 2018, Piccolino 1997, Prindle et al. 2015), but also for intracolony or interspecies communication (Beagle and Lockless 2015, Clarke et al. 2013, Prindle et al. 2015). The cell morphology and growth mode of filamentous fungi provides, in theory, an ideal system for electrical signalling, but the use of traditional electrophysiological methods for measuring these signals is extremely challenging (Adamatzky 2022). With integrated electronics, microfluidic chips are envisaged to be beneficial to further investigate these phenomena at the hyphal level.

While most microfluidic devices are made from mainly PDMS, other polymers or glass, the versatility of design possibilities goes far beyond these materials. Some groups set out to integrate metals into their polymer-based devices. Singh et al. (2017), for instance, coated microchannels with a thin layer of gold. Thus, they designed a microfluidic platform to capture bacteria and fungi efficiently, based on the affinity of gold towards thiol and amine groups, which are present in the microbes’ cell walls. The applications of such a device range from immobilization for imaging to concentration of target microbes from mixed and real-life samples for further analyses or diagnostics. The primary property of metals, however, relates to their electrical conductivity. Duarte et al. (2022) built an array of nanoelectrodes into a PDMS microfluidic device. These electrodes allowed for both dielectrophoretic trapping, as well as subsequent detection and identification of spores from the phytopathogenic fungus *Sclerotinia sclerotiorum*. The possibility to combine visual, imaging studies with electrical measurements opens entirely new avenues. With the integration of microfluidic technologies with electronic components, electrophysiological measurements on fungi and their partners could be performed and further insights into the fungi’s role as the ‘brain’ of the soil gained.
